# Translation-dependent unwinding of stem–loops by UPF1 licenses Regnase-1 to degrade inflammatory mRNAs

**DOI:** 10.1093/nar/gkz628

**Published:** 2019-07-22

**Authors:** Takashi Mino, Noriki Iwai, Masayuki Endo, Kentaro Inoue, Kotaro Akaki, Fabian Hia, Takuya Uehata, Tomoko Emura, Kumi Hidaka, Yutaka Suzuki, Daron M Standley, Mariko Okada-Hatakeyama, Shigeo Ohno, Hiroshi Sugiyama, Akio Yamashita, Osamu Takeuchi

**Affiliations:** 1 Department of Medical Chemistry, Graduate School of Medicine, Kyoto University, Kyoto 606-8501, Japan; 2 Department of Chemistry, Graduate School of Science, Kyoto University, Kitashirakawa-oiwakecho, Sakyo-ku, Kyoto 606-8502, Japan; 3 Institute for Integrated Cell-Material Sciences (iCeMS), Kyoto University, Yoshida-ushinomiyacho, Sakyo-ku, Kyoto 606-8501, Japan; 4 Department of Computer Science and Systems Engineering, Faculty of Engineering, University of Miyazaki, Miyazaki 889-2192, Japan; 5 Laboratory of Functional Genomics, Department of Medical Genome Sciences, Graduate School of Frontier Sciences, The University of Tokyo, Chiba 277-8562, Japan; 6 Department of Genome Informatics, Genome Information Research Center, Research Institute for Microbial Diseases (RIMD), Osaka University, Osaka 565-0871, Japan; 7 Laboratory for Integrated Cellular Systems, RIKEN Center for Integrative Medical Sciences, Yokohama 230-0045, Japan; 8 Laboratory of Cell Systems, Institute for Protein Research, Osaka University, Osaka 565-0871, Japan; 9 Department of Molecular Biology, Yokohama City University School of Medicine, Kanagawa 236-0004, Japan

## Abstract

Regnase-1-mediated mRNA decay (RMD), in which inflammatory mRNAs harboring specific stem–loop structures are degraded, is a critical part of proper immune homeostasis. Prior to initial translation, Regnase-1 associates with target stem–loops but does not carry out endoribonucleolytic cleavage. Single molecule imaging revealed that UPF1 is required to first unwind the stem–loops, thus licensing Regnase-1 to proceed with RNA degradation. Following translation, Regnase-1 physically associates with UPF1 using two distinct points of interaction: The Regnase-1 RNase domain binds to SMG1-phosphorylated residue T28 in UPF1; in addition, an intrinsically disordered segment in Regnase-1 binds to the UPF1 RecA domain, enhancing the helicase activity of UPF1. The SMG1-UPF1–Regnase-1 axis targets pioneer rounds of translation and is critical for rapid resolution of inflammation through restriction of the number of proteins translated by a given mRNA. Furthermore, small-molecule inhibition of SMG1 prevents RNA unwinding in dendritic cells, allowing post-transcriptional control of innate immune responses.

## INTRODUCTION

Control of gene expression by mRNA degradation plays a critical role in the regulation of various biological processes, including immune reactions, responses to cellular stresses and maintenance of homeostasis (1,2). In mammals, mRNA degradation is mediated by a set of RNases, whose activity is tightly regulated in order to control the abundance of specific mRNAs. There are several known modes of regulation of RNase-mediated mRNA decay. One mode affects the recruitment of RNases to the mRNAs targeted for degradation. Deadenylation and subsequent decay of mRNAs is controlled by recruitment of the CCR4–NOT deadenylase complex which targets mRNAs via RNA binding proteins (RBPs) such as tristetraprolin and Roquin or microRNAs (3,4). Nonsense-mediated mRNA decay (NMD) is also known to be regulated by the recruitment of the endoribonuclease SMG6 or the CCR4–NOT complex via SMG5-7 to aberrant mRNAs by binding with the helicase UPF1 (5–9). In addition to such recruitment-induced activation of RNases, dimerization of RNases can act as a switch to initiate mRNA digestion. For instance, the endoribonuclease IRE1α cleaves *XBP1* pre-mRNA upon endoplasmic reticulum (ER) stress by homodimerization to induce splicing and production of XBP1 protein (10–12). RNase L is another example of an RNase activated by its homodimerization in response to virus infection (13,14). Thus, control of RNA degradation by altering the conformation or oligomerization state of the RNase is a well-established mode of regulation. On the other hand, recent studies have revealed that mRNA structures themselves influence their post-transcriptional fate, and that these structures dynamically change in response to stimuli, such as virus infection (15,16). However, the relationship between structural changes of mRNAs and their degradation in the regulation of cellular responses has not been clarified.

Regnase-1 (also known as Zc3h12a and Mcpip1) is an endoribonuclease essential for the maintenance of immune and iron homeostasis (17,18). Regnase-1 suppresses inflammation by promoting decay of immune-related mRNAs, including *Interleukin 6* (*IL6*), *IL12B, PTGS2 and NFKBIZ*—but not *NFKBIA*—in innate immune cells following stimulation with Toll-like receptor (TLR) ligands such as lipopolysaccharide (LPS) and IL-1β (17,19,20). Macrophages from *Regnase-1*-deficient mice express the target mRNAs abundantly, and produce elevated amounts of proinflammatory cytokines, which contributes to the development of severe autoimmunity. For these reasons Regnase-1 activity must be tightly regulated at multiple levels. Crosslinking and immunoprecipitation sequencing (CLIP-Seq) analysis revealed that Regnase-1 recognizes mRNAs harboring stem–loop structures with a pyrimidine–purine–pyrimidine (Py–Pu–Py) loop sequence present in the 3′ untranslated regions (3′ UTRs) of target mRNAs as *cis*-elements (21). The stem–loop structure is recognized not only by Regnase-1 but also Roquin, an RNA-binding protein critical for the maintenance of immune homeostasis (20,22,23). However, Regnase-1 and Roquin function by spatiotemporally distinct mechanisms. Regnase-1 and Roquin tend to control mRNAs at the early and late phase of inflammation, respectively. Whereas Roquin destabilizes translationally inactive mRNAs by recruiting the CCR4–NOT complex in processing-bodies (PBs) and stress granules (SGs), Regnase-1 localizes to the ER and ribosomes, destabilizes translationally active mRNAs and requires the RNA helicase, UPF1, which is also essential for NMD (21). In NMD, phosphorylated UPF1 is recruited to nonsense mRNAs and interacts with SMG6 or the SMG5-7 complex (7). The helicase/ATPase activity of UPF1 is required for disassembly of messenger ribonucleoproteins (mRNPs) undergoing NMD (24), and also for its preferential release from non-target mRNAs (25). Although it has been demonstrated that Regnase-1-mediated decay (RMD) depends on UPF1 in a translation-dependent manner, the role of this interaction in the regulation of Regnase-1 activity has not yet been elucidated.

In this study, we found that structural changes in the stem–loop RNA mediated by UPF1 function as the structural switch for execution of RMD. The UPF1–Regnase-1 interaction is induced subsequent to a pioneer round of translation, whereas Regnase-1 recruitment to target mRNAs occurs irrespective of translation termination. Mutational analysis revealed that the phosphorylation of UPF1 at T28 by the kinase SMG1 following translation is required for stable interaction between UPF1 and Regnase-1. Furthermore, inhibition of SMG1 kinase activity with a specific inhibitor induces DC maturation and potentiates T cell-stimulatory activity. Collectively, this study reveals a heretofore undescribed mechanism of mRNA decay triggered not by conformational changes in the RNase but in the RNA, and thus represents an additional level of RNase-mediated control of inflammatory responses.

## MATERIALS AND METHODS

### Mice

Mice deficient in Regnase-1 have been described (17). *Sanroque* mice were kindly provided by Dr Masakazu Hattori (Kyoto University). C57BL/6J and BALB/c mice were purchased from CREA Japan. All animal experiments were done with the approval of the Animal Research Committee of the Institute for Frontier Life and Medical Sciences and Graduate School of Medicine, Kyoto University.

### Cell culture

HeLa cells and HEK293T cells were purchased from ATCC and maintained in DMEM (Nacalai Tesque) supplemented with 10% (vol/vol) FBS (Gibco), 100 U/ml of penicillin (Nacalai Tesque), 100 μg/ml of streptomycin (Nacalai Tesque) and 50 μM β-mercaptoethanol (Nacalai Tesque). Tet-off HEK293T cells were purchased from Clontech and maintained in α-MEM (Nacalai Tesque) supplemented with 10% (vol/vol) FBS (Clontech), 50 μM β-mercaptoethanol and 100 μg/ml G418 (Nacalai Tesque).

*Regnase-1* ^–/–^ HeLa cells were generated by CRISPR-Cas9 gene editing as previously reported method (26). Briefly, to delete target genes, SpCas9-expressing HeLa cells were lentivirally transduced with a plasmid carrying respective sgRNA (target sequence for human Regnase-1, 5′-TGAGACCAGTGGTCATCGAT-3′) and selected with puromycin (1 μg/ml) for 7 days. Selected cells were pooled and validated for gene deletion by immunoblot analysis.

Primary MEFs were prepared from wild-type, *Regnase-1*^–/–^, *Roquin*^san/san^ mouse embryos at embryonic day 13.5 and maintained in DMEM supplemented with 10% (vol/vol) FBS (Gibco) and 50 μM β-mercaptoethanol.

Peritoneal macrophages were prepared from mice 3 d after intraperitoneal injection of 4% (vol/vol) thioglycollate medium (2 ml) (Sigma) and were maintained in RPMI-1640 medium (Nacalai Tesque) supplemented with 10% (vol/vol) FBS (Gibco) and 50 μM β-mercaptoethanol.

For preparation of bone marrow–derived macrophages (BMMs), bone marrow cells were isolated from wild-type, *Regnase-1*^–/–^ or *Roquin*^san/san^ mice and cultured in macrophage growth medium (RPMI-1640 medium supplemented with 10% (vol/vol) FBS (Gibco), 50 μM β-mercaptoethanol, 100 U/ml of penicillin, 100 μg/ml of streptomycin and 20 ng/ml of macrophage colony-stimulating factor (BioLegend)). After 5 days, cells were washed once and cultivated for 2 days with macrophage growth medium, then cells were collected for further analysis.

For preparation of bone marrow–derived dendritic cells (BMDCs), bone marrow cells were isolated from wild-type or *Regnase-1*^–/–^ mice and cultured in dendritic cell growth medium (RPMI-1640 medium supplemented with 10% (vol/vol) FBS, 50 μM β-mercaptoethanol, 100 U/ml of penicillin, 100 μg/ml of streptomycin, 250 ng/ml amphotericin B and 10 ng/ml of murine granulocyte-macrophage colony-stimulating factor (PeproTech). Every 2 days, non-adherent cells were discarded and remaining cells were fed with fresh dendritic cell growth medium. At day 6, loosely adherent cells were harvested and used for indicated assays.

## METHOD DETAILS

### DNA and siRNA transfection

Cells were transfected with plasmid DNAs through the use of Lipofectamine 2000 or Lipofectamine LTX (Thermo Fisher Scientific) according to the manufacturer's recommendations.

For siRNA-mediated knockdown, cells were transfected through the use of Lipofectamine RNAiMAX (Thermo Fisher Scientific) or MISSION siRNA Transfection Reagent (Sigma). The siRNAs used in this study were synthesized by Thermo Fisher Scientific and the following siRNA target sequences were used: hUPF1, 5′-GAUGCAGUUCCGCUCCAUUdTdT-3′; hSMG1, 5′-GUGUAUGUGCGCCAAAGUAdTdT-3′; hRegnase-1, 5′-GUGUCCCUAUGGAAGGAAAdTdT-3′; hRoquin-1, 5′-GAUCGAGAGUUACUAUCCAdTdT-3′.

### Plasmid construction and reagents

The cDNAs of mouse Regnase-1 (Zc3h12a), Regnase-1 mutants and mouse Il6 have been described (17). The cDNAs of mouse Roquin-1 (Rc3h1) was kindly provided by Georg Stoecklin (German Cancer Research Center). The cDNAs of Regnase-1, Roquin and human UPF1 were ligated to the vector pFlag-CMV2 (SIGMA), pcDNA3.1(+) (Invitrogen), pEGFP-C1 (Clontech), pmCherry-C1 (Clontech) and pSR (7) for mammalian expression. The cDNA of *Il6* CDS-3′ UTR was inserted in pTREtight vector (Clontech). The 3′ UTR cDNAs of a set of genes were inserted in the pGL3-promoter (Promega).

Mammalian expression vectors for wild-type human UPF1 and its mutants [pSR-HA-hUPF1-WT (amino acids 6–1118), pSR-HA-hUPF1-WT^R^ (amino acids 6–1118), pSR-HA-hUPF1-ΔNT (amino acids 64–1118), pSR-HA-hUPF1-ΔCT (amino acids 6–1027), pSR-HA-hUPF1-ΔNCT (amino acids 64–1027), pSR-HA-hUPF1-T28A, pSR-HA-hUPF1–4SA (SSSS1073/1078/1096/1116AAAA), pSR-HA-hUPF1-T28E] were previously described (7,21). siRNA resistance mutants of wild type human SMG1 and its mutants [pSR-Flag-hSMG1-WT^R^ and pSR-Flag-hSMG1-D2331A^R^] were generated by standard methods. pSR-HA-hUPF1-ΔCH (amino acids 295–914), pSR-HA-hUPF1-(428–964), pSR-HA-hUPF1-(6–429), pSR-HA-hRIG-I-WT (amino acids 2–925), pEFh-SBP-GFP-hUPF1-(295–914), pEFh-SBP-GFP-hUPF1-(416–914) and pEFh-SBP-GFP-hUPF1-(610–914) were made by standard cloning procedures. The cDNAs of human UPF1 (6–429) were ligated to the vector pEGFP-C1 (Clontech) for mammalian expression. The targeted mutations into Regnase-1 and UPF1 genes were introduced by QuikChange Lightning Site-Directed Mutagenesis Kit (Agilent). pCMV-Myc-hUPF1-G495R/G497E was kindly provided by Lynne E. Maquat (University of Rochester).

LPS derived from Salmonella minnesota, Pam_3_CSK_4_, R848, CpG oligonucleotide ODN1668 and puromycin were purchased from Invivogen; Recombinant cytokines were purchased from R&D Systems; Anisomycin, 5,6-dichloro-1-beta-D-ribofuranosylbenzimidazole (DRB), Emetine and actinomycin D were purchased from Sigma; α-Amanitin was purchased from Wako; SMG1 inhibitor (referred to as ‘Compound 11J’ in (27)) was purchased from TLC Pharmaceutical Standards; Compund 11J (SMG1 inhibitor), KU-60019 (ATM inhibitor), VE-821 (ATR inhibitor) and NU7441 (DNA-PK inhibitor) were purchased from Selleckchem.

### Immunoblot analysis

Whole-cell extracts were prepared in lysis buffer (1% (vol/vol) Nonidet P-40, 0.1% (wt/vol) SDS, 1% (wt/vol) sodium deoxycholate, 150 mM NaCl, 20 mM Tris–HCl, pH 8.0, 10 mM EDTA and Complete Mini Protease Inhibitor Cocktail without EDTA (Roche)) and suspended in SDS sample buffer (50 mM Tris–HCl, pH 6.8, 2% (wt/vol) SDS, 5% (vol/vol) β-mercaptoethanol, 10% (vol/vol) glycerol and bromophenol blue). Proteins were boiled for 5 min at 95°C, resolved on polyacrylamide gels (e-PAGEL; ATTO) and transferred onto 0.2 μm pore size Immun-Blot PVDF membranes (Bio-Rad). Membranes were incubated with indicated primary antibodies and HRP-coupled secondary antibodies (NA9310 and NA9340; GE Healthcare). The following primary antibodies were used for immunoblot analysis: antibody to Flag (F3165 and F7425; Sigma), HA (H6908 and H3663; Sigma), GFP (ab1218 and ab290; Abcam), mCherry (ab125096 and ab183628; Abcam), SBP (ab119491; Abcam), CBP80 (A301; Bethyl Laboratories), eIF4E (#9742; CST), PABPC (#4992; CST), SMG1 (A300-393A; Bethyl Laboratories), IκBα (sc-371; Santa Cruz), Roquin-1 (A300-514A; Bethyl Laboratories), Roquin-2 (ab99090; Abcam), Ptgs2 (ab15191; Abcam) and β-actin (sc-1615; Santa Cruz). Rabbit IκB-z (NFKBIZ) polyclonal antibody was kindly provided by Tatsushi Muta (Tohoku University). Rabbit Regnase-1 antibody was described (19). Rabbit anti-UPF1 affinity antibody, rabbit anti-P-T28-UPF1 antibody and mouse anti-P-S1078/S1096-UPF1 monoclonal antibody (cone 8E6) were previously described (7,28,29). The anti-P-S1078/S1096-UPF1 antibody recognizes both phosphorylated S1078 and S1096 residues (7). Membranes were treated with Luminata Forte Western HRP substrate (Millipore) and luminescence was detected with a luminescent image analyzer (Amersham Imager 600; GE Healthcare).

### Northern blotting

Total RNAs were isolated using ISOGEN II (Wako) or Trizol (Invitrogen), electrophorated, blotted to Hybond-N+ (GE healthcare) and hybridized with the probes for *Il6* and *ACTIN* as previously described (17). The membranes were exposed to an imaging plate and analyzed by BAS-5000 imaging analyzer (Fuji Film). Primers used for producing probes were shown in Supplementary Table S3.

### Quantitative PCR analysis

Total RNAs were isolated using ISOGEN II (Wako) or Trizol (Invitrogen) and reverse-transcribed using ReverTra Ace (Toyobo) according to the manufacturer's instructions. For quantitative PCR, cDNA fragments were amplified through the use of universal SYBR Select Master Mix (Thermo Fisher Scientific). Fluorescence was detected with a StepOne Real-Time PCR System (Applied Biosystems). Primers used for qPCR were shown in Supplementary Table S3. The abundance of mRNA of each expressed gene was normalized to that of β-Actin.

### Protein IP

HeLa cells transfected with the indicated siRNA and expression plasmids were lysed in lysis buffer (0.1% (vol/vol) Nonidet P-40, 150 mM NaCl, 10 mM Tris–HCl, pH 8.0 and Complete Mini Protease Inhibitor Cocktail without EDTA (Roche)). The indicated antibodies, anti-Flag antibody (F3165; Sigma) or anti-GFP antibody (ab1218; Abcam), bound to protein G magnetic beads (Invitrogen) was incubated for 3 h at 4°C with lysates. Beads were washed three times with lysis buffer and suspended in SDS sample buffer (50 mM Tris–HCl, pH 6.8, 2% (wt/vol) SDS, 5% (vol/vol) β-mercaptoethanol, 10% (vol/vol) glycerol and bromophenol blue). Samples were boiled for 5 min at 95°C and analyzed by western blot with the indicated antibodies.

### RNA IP

HeLa cells and Tet-off 293T cells transfected with the indicated siRNA and expression plasmids were lysed in RNA IP lysis buffer (0.1% (vol/vol) Nonidet P-40, 150 mM NaCl, 20 mM Tris–HCl, pH 7.5, Complete Mini Protease Inhibitor Cocktail without EDTA (Roche) and 0.2 U/ml RNasin (Promega)). The indicated antibodies, anti-Flag antibody (F3165; Sigma), normal rabbit IgG (MLB), anti-αCBP80 mouse monoclonal clone 38A1 antibody or anti-eIF4E antibody (RN001P; MLB), bound to protein G magnetic beads (Invitrogen) was incubated for 3 h at 4°C with lysates and beads were washed three times with RNA IP lysis buffer. Anti-αCBP80 antibody was kindly provided by Mutsuhito Ohno in Kyoto University and details of the generation of anti-αCBP80 antibody is described (30). RNAs were eluted from the beads using Trizol reagent (Invitrogen) according to the manufacturer's instructions and analyzed by RT-qPCR. Proteins were eluted from the beads using SDS sample buffer (50 mM Tris–HCl, pH 6.8, 2% (wt/vol) SDS, 5% (vol/vol) β-mercaptoethanol, 10% (vol/vol) glycerol and bromophenol blue) and analyzed by Western blot with the indicated antibodies.

### RNA immunoprecipitations (RIP)-sequencing analysis

RNA immunoprecipitation was performed with Regnase-1-WT and -KO HeLa cells in biological duplicate as described above. The quality of RNA was analyzed by the Bioanalyzer Nano 6000 chip (Agilent Technologies). RNA library was prepared using Sureselect Strand mRNA kit (Agilent Technologies) and sequenced on a HiSeq 3000 system (Illumina) according to the manufacturer's instructions. Approximately 52–70 million RNA reads were obtained. The resulting set of trimmed reads were then mapped against the human genome (hg19; NCBI). Analysis of enrichment of mapped reads in the Regnase-1 RIP KO samples vs WT samples for CBP80, eIF4E and total input RNA were performed using the R package, edgeR (31), as follows. The count of mapped reads to each gene was used as input for the method. The coverage of each sample and the dispersions were estimated from the read count data using the functions provided in the package. Finally, differential tag counts for each gene between the RIP KO and WT samples for CBP80, eIF4E and total input RNA were estimated based on a negative binomial distribution respectively. Genes with an adjusted p value for enrichment in the RIP samples <1e–2 were regarded as candidate Regnase-1 targets.

### Analysis of overlap with between Regnase-1 targets and enrichments in CBP80/eIF4E RIP-seq

The overlap between the targets of Regnase-1 and those from the Regnase-1 RIP CBP80, eIF4E and total input RNA experiments were evaluated using three measures. First, we employed the Gene Set Enrichment Analysis (GSEA) methodology, as described (32). In this case, we obtained a set of genes S, defined from a previous publication (21). On the other hand, a ranked list L was obtained by sorting the genome‐wide set of genes by their enrichment in the RIP-seq CBP80, eIF4E and total input RNA samples respectively. The distribution of S in L was evaluated by calculating an Enrichment Score (ES) and the estimation of a significance level for ES by 1,000 random permutations, as described (32). The P value of enrichment of S in the top of L was estimated as the fraction of permuted samples.

### mRNA Decay assay

Doxycycline (1 μg/ml) (Sigma) or actinomycin D (1 μg/ml) was added to the medium for the indicated time intervals before harvesting Tet-off HEK293T cells or HeLa cells, respectively. Total RNA was isolated using ISOGEN II (Wako) or Trizol (Invitrogen). mRNA levels were determined either by northern blot or by RT-qPCR analysis.

### Luciferase assay

HeLa cells and HEK293T cells were transfected with luciferase reporter plasmid pGL3 containing the 3′ UTR of indicated genes, together with expression plasmid for Regnase-1 or empty (control) plasmid. After 24 h of cultivation, cells were lysed, and luciferase activity in lysates was determined with the Dual-Luciferase Reporter Assay system (Promega). The gene encoding Renilla luciferase was transfected simultaneously as an internal control.

### Recombinant proteins

Recombinant Regnase-1 protein has been previously described (17,21). For preparation of HA-UPF1 protein, HeLa cells were transfected with expression vectors for HA-UPF1 and lysed in lysis buffer (0.1% (vol/vol) Nonidet P-40, 150 mM NaCl, 10 mM Tris–HCl, pH 8.0 and Complete Mini Protease Inhibitor Cocktail without EDTA (Roche)). The HA-UPF1 protein was purified by HA tagged protein purification kit (MBL) according to the manufacturer's protocol.

### *In vitro* RNA cleavage assay

*In vitro* RNA cleavage assay has been previously described (17,21). Briefly, 5′-[^32^P]-labeled RNAs (0.25 pmol) were mixed with recombinant Regnase-1 protein (0.75 pmol) (21) and HA-UPF1 protein in a cleavage buffer (25 mM HEPES (pH 7.5), 50 mM potassium acetate, 5 mM DTT, 5 mM magnesium acetate, 2 mM ATP and 0.2 U/ml RNasin (Promega)) at 37°C. The cleaved RNAs were analyzed by denaturing TBE–urea gel (Invitrogen) and autoradiography. For two-step reaction for UPF1 and Regnase-1, 5′-[^32^P]-labeled RNAs were incubated with HA-UPF1 at 37°C for 10 min, immediately cooled at 4°C and then immediately (within 5 min) started the reaction with Regnase-1. The UPF1-mediated change in the RNA structure was kept just before the start of incubation with Regnase-1. The RNAs were analyzed by denaturing TBE–urea gel and native PAGE electrophoresis.

### ATPase assay

The proteins were incubated in a reaction buffer (25 mM HEPES (pH7.5), 50 mM potassium acetate, 5 mM DTT, 5 mM magnesium acetate, 2 mM ATP, 0.01 pmol/μl RNA and 0.2 U/ml RNasin) at 37°C and phosphates from UPF1-mediated hydrolysis of ATP were measured by BIOMOL GREEN reagent (Enzo Life Sciences Inc).

### Unwinding assay

5′-[^32^P]-labeled RNAs (0.25 pmol) were incubated with Regnase-1 (0.75 pmol) and HA-UPF1 in the cleavage buffer without Mg^2+^, purified by QIAquick Nucleotide Removal Kit (QIAGEN, 28304) at 4°C. The RNAs were analyzed by native PAGE electrophoresis and autoradiography.

### GST pull-down assay

GST-Regnase-1 was incubated with HA-UPF1 in a reaction buffer [0.1% NP-40, 10 mM Tris–HCl (pH8.0), 150 mM NaCl, 5 mM Mg(OAc)_2_, 5 mM ZnCl_2_, 1 mM DTT, 10% glycerol] for 1 h at r.t., immunoprecipitated with Glutathione Sepharose for 1 h at 4°C. The sepharose beads were washed three times with the reaction buffer and the complex was analyzed by western blot.

### High-speed atomic force microscopy (AFM) observation

#### DNA frame formation

DNA frame used in the experiment was designed and prepared as previously reported method (33,34). The DNA frame was assembled in a 20 μl solution containing 10 nM of M13mp18 single-stranded DNA (Tilibit nanosystems, Munich, Germany), 40 nM of staple strands (226 strands), 20 mM Tris–HCl (pH 7.6), 1 mM EDTA, and 10 mM MgCl_2_. The mixture was annealed from 85 to 15°C at a rate of −1.0°C/min.

#### Preparation of RNA strands

RNA strands containing specific stem/hairpin or non-specific sequence were prepared using *in vitro* transcription. The DNA template containing T7 promotor and target sequence was prepared by PCR amplification. Transcription was performed at 42°C for 2 h in a 40 μl solution containing 0.1 μM DNA template, T7 RNA polymerase (100 units, Takara, Kyoto, Japan), and 1 mM NTP. The reaction mixture were purified using Qiagen RNA purification kit.

#### Incorporation of RNA strands into the DNA frame

For incorporation of the RNA strands, two DNA connector strands were used to hybridize with RNA strand and fix to the DNA frame. After annealing, the pre-assembled DNA/RNA hetero duplex were incorporated into the DNA frame by annealing the mixture from 40 to 15°C at a rate of −1.0°C/min using a thermal cycler. The sample was purified by gel-filtration (GE sephacryl-400, GE Healthcare Japan, Tokyo, Japan).

#### Reaction with Regnase-1 and UPF1 in the DNA frame

For AFM observation, DNA frame (3.8 nM) with substrate RNA was incubated with Regnase-1 (125 nM) and UPF1 in a reaction buffer (25 mM HEPES (pH7.5), 50 mM potassium acetate, 5 mM DTT, 5 mM magnesium acetate, 2 mM ATP) at 30°C for 10 min. After the incubation, 2 μl of the three times diluted sample was deposited onto a freshly cleaved mica disc. After 5 min incubation, the absorbed sample was washed with 2 μl of the imaging buffer six times and imaged in the same buffer.

#### AFM imaging

Imaging was performed using a high-speed AFM (Nano Live Vision, RIBM, Tsukuba, Japan). The sample was imaged in the imaging buffer solution at ambient temperature using an ultra-short cantilever (USC-F1.2-k0.15, Nanoworld, Switzerland). These cantilevers have a spring constant of 0.15 N/m with a resonant frequency of 1200 kHz in water. The 320 × 240 pixel images were obtained at a scan rate of 0.2 frames per second (fps).

### Mathematical model

We constructed a mathematical model based on the assumption that the relationship between mRNA and protein levels of *PTGS2* in experiment can be approximated by mass action and Hill equations (Figure 6G and H). The equations of the mathematical model are described as follows:

$$\begin{equation*}\begin{array}{@{}*{3}{l}@{}} {\frac{{d[CmRNA]}}{{dt}}}& = &{{k_1}\ \cdot signal(t) - {k_2}[CmRNA]}\\ {}&{}&{\ - {k_3}[CmRNA] - {k_4}[Reg1][CmRNA]} \end{array}\end{equation*}$$

$$\begin{equation*}\begin{array}{@{}*{3}{l}@{}} {\frac{{d[EmRNA]}}{{dt}}}& = &{{k_2}\ [CmRNA] - {k_5}[EmRNA]}\\ {}&{}&{ - {k_6}[Reg1][EmRNA] - {k_7}[Roq][EmRNA]} \end{array}\end{equation*}$$

$$\begin{equation*}\begin{array}{@{}*{3}{l}@{}}{\frac{{d[Protein]}}{{dt}}}& = &{{k_8}\ [CmRNA] + \frac{{{k_9}{{[EmRNA]}^h}}}{{k{m^h} + {{[EmRNA]}^h}}}}\\ {}&{}&{ - {k_{10}}[Protein]} \end{array}\end{equation*}$$

where *CmRNA* is the amount of CBP80-bound mRNA, *EmRNA* is the amount of eIF4E-bound mRNA, *Protein* is the amount of a protein, *Regnase-1* is the Regnase-1 activity, *Roq* is the Roquin activity, *signal*(*t*) is stimulus-induced gene expression, *k_i_* (*i* = 1,…,10) is reaction rates, *km* is half maximal effective concentration of translation from *EmRNA*, and *h* is Hill coefficient. *signal*(*t*) is described as follows (35):

$$\begin{equation*}signal{\rm{\ (}}t{\rm{)}} = \left\{ {\begin{array}{@{}*{1}{l}@{}} {{s_{base}}{\rm{\ }}\left( {{\rm{if\ }}\,0 \le t \le {t_{delay}}} \right),}\\ {\frac{{{s_{input}} - {s_{base}}}}{{{t_{raise}}}}\left( {t - {t_{delay}}} \right) + {s_{base}}{\rm{\ }}\left( {{\rm{if\ }}{t_{delay}} < t \le {t_{raise}} + {t_{delay}}} \right),}\\ {{s_{input}}{\rm{\ }}\left( {{\rm{if\ }}{t_{raise}} + {t_{delay}} < t \le {t_{pulse}} + {t_{raise}} + {t_{delay}}} \right),}\\ {\left( {{s_{input}} - {s_{late}}} \right) \times {\rm{\ exp}}\left( { - \frac{{t - {t_{pulse}} - {t_{raise}} - {t_{delay}}}}{{{t_{delay}}}}} \right)}\\ { + {s_{late}}{\rm{\ }}\left( {{\rm{if\ }}t >{t_{pulse}} + {t_{raise}} + {t_{delay}}} \right).} \end{array}} \right.\end{equation*}$$

*Regnase-1* and *Roq* are employed as interpolation of experimental data using the function *pchip* of MATLAB R2014a (MathWorks, Natick MA) (Supplementary Figure S6B). First, we fit the parameter values of *k_1_*,…, *k_7_* against mRNAs for PTGS2 and NFKBIZ in experiments using different parameter values in *signal*(*t*). Then, the other parameters were fitted against proteins of PTGS2 and NFKBIZ in experiments. The parameter values are listed in Tables S1 and S2. Simulations were performed using the function *ode15s* of MATLAB R2014a (MathWorks, Natick MA).

### Sensitivity analysis

The single parameter sensitivity of each reaction is defined by

$$\begin{equation*}{s_i} \left({q({\bf p}),{p_i}} \right) = \frac{{\partial \ln q({\bf p})}}{{\partial \ln {p_i}}}\ = \frac{\partial q({\bf p})\,{p_{i}}}{{\partial {p_i}}\,{q({\bf p})}}\end{equation*}$$

where *q* is the target function, **p** = (*p_1_*,*p_2_*,…,*p_n_*) is the kinetic parameter vector, and *p_i_* is the *i*th kinetic parameter. We used integrals of time courses of eIF4E-mRNA and protein as target function in Supplementary Figure S6G and H, respectively, and the sensitivity for 1% change of each kinetic parameter.

### Structure modelling

The structural model of phosphothreonine (P-Thr) docked to the Regnase-1 RNase domain (134–296) via K257R258 was constructed using Autodock Vina version 1.1.2 for MacOSX (36). The P-Thr coordinates were downloaded from SwissSideChain (37) and the Regnase-1 coordinates were taken from Protein Data Bank entry 3V33. The docking was performed within a sphere of radius 10Å centered at residue Arg 254 using default parameters and the lowest-energy pose was retained.

### ELISA for culture supernatants

BMDCs from C57BL/6J mice were treated with or without 0.3 μM SMG1 inhibitor and stimulated with 10 ng/ml Pam_3_CSK_4_ (Invivogen), 100 ng/ml LPS (Invivogen), 100 nM R848 (Invivogen) or 100 nM ODN1668 (Invivogen) for 24 h and cytokine levels in culture supernatants were measured by cytokine-specific ELISA kits (affymetrix eBioscience) following manufacturer's protocols.

### Flow cytometry

BMDCs were cultured with 1 ng/ml Pam_3_CSK_4_ (Invivogen) and/or 0.3 μM SMG1 inhibitor for 48 hr, stained with indicated antibody for 30 min at 4 °C and washed twice with FACS buffer (0.5% (wt/vol) BSA and 2 mM EDTA in PBS). The following antibodies were used for flow cytometric analysis: anti-CD11c (117308; BioLegend), anti-CD40 (124612; BioLegend) and anti-CD80 (104713; BioLegend). Data were acquired by FACSVerse and LSRFortessa X-20 (BD Biosciences), and analyzed using FlowJo.

### Mixed lymphocyte reaction

BMDCs from C57BL/6J mice (CREA Japan) were cultured with 1 ng/ml Pam_3_CSK_4_ (Invivogen) and/or 0.3 μM SMG1 inhibitor for 48 h, washed, irradiated at a dose of 30 Gy and plated at 3-fold serial dilutions in 96-well round-bottom plates. These stimulator BMDCs were cocultured for 3 days with 5 × 10^4^ cells/well of splenic CD4^+^ T cells from BALB/c mice (CREA Japan). CD4^+^ T cells were isolated by using autoMACS with CD4(L3T4) microbeads (Miltenyi Biotec). [^3^H]Thymidine was added for the last 16 h and [^3^H]thymidine incorporation was measured using a microplate scintillation counter, TopCount NXT (Packard).

### Quantification and statistical analysis

Unless otherwise indicated, data are presented as means ± SD (*n* = 3). Statistical significance was calculated with a Student's *t* test. Significance was accepted at the level of *P* < 0.05 (*), *P* < 0.01 (**), or *P* < 0.001 (***). Western blot images were quantitated by Amersham Imager 600 (GE Healthcare) with ImageQuant TL software (GE Healthcare). Autoradiographic images were quantitated by BAS-5000 imaging analyzer (Fuji Film) with Multi Gauge software (Fuji Film).

## RESULTS

### Regnase-1 interacts with, but does not degrade, untranslated stem–loop mRNAs

**Figure 1.**
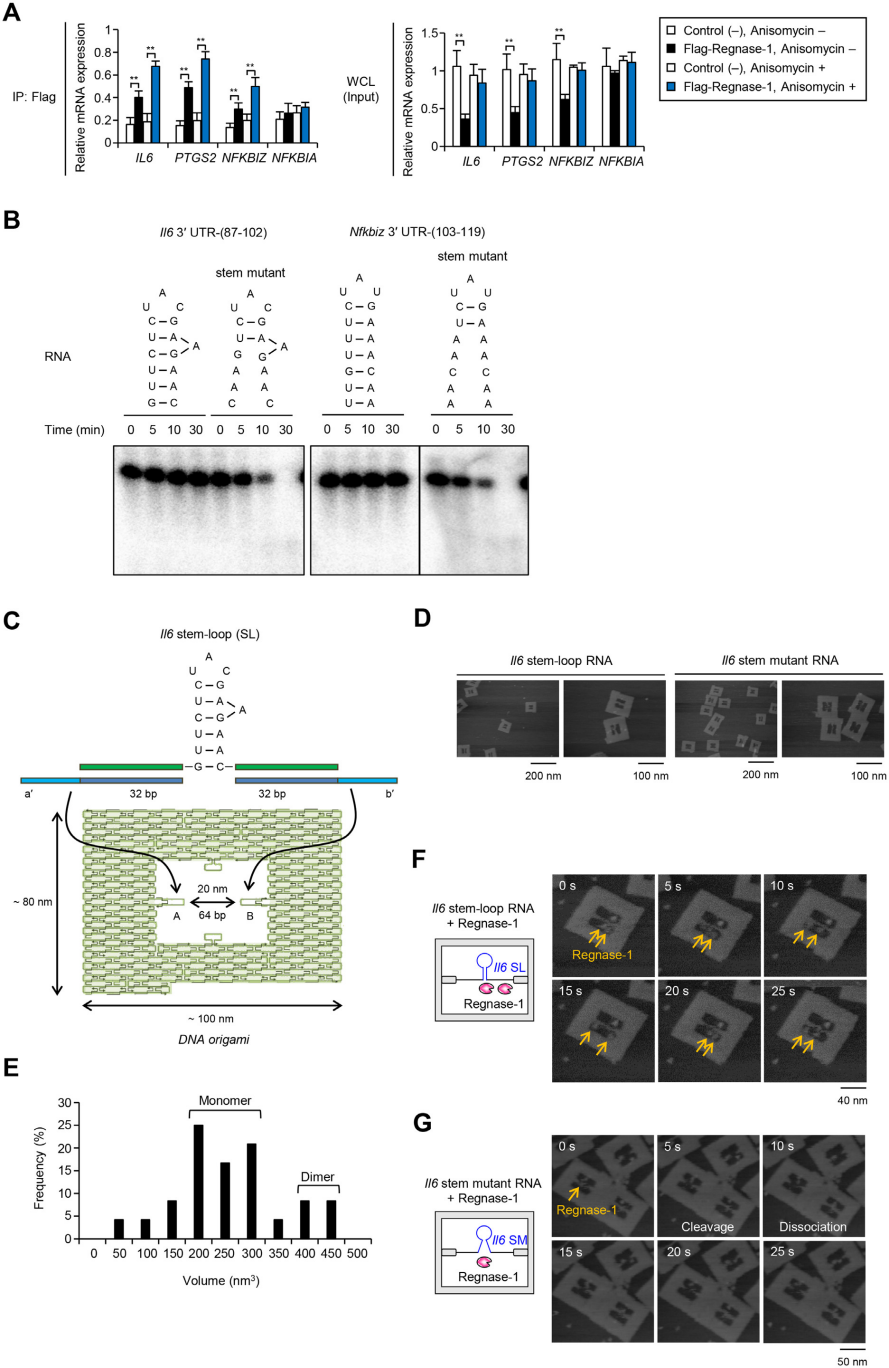
Regnase-1 does not degrade stem–loop RNA on target mRNAs. (**A**) mRNAs associated with Regnase-1 in HeLa cells transfected with indicated expression plasmids, stimulated with IL-1β (10 ng/ml) and treated with anisomycin (10 μg/ml) for 2 h were analyzed by RIP-qPCR. (**B**) The stem–loop RNAs are not cleaved by Regnase-1 *in vitro* cleavage assay. Indicated 5′-^32^P-labeled RNAs were incubated with Regnase-1 for indicated time, and analyzed by TBE–urea gel electrophoresis. (**C**) Illustration of DNA origami frame with *Il6* 3′ UTR-(87–102) stem–loop RNA. (**D**) AFM images of complex of DNA frame with *Il6* RNAs. (**E**) The volume of Regnase-1 particles was calculated from AFM imaging by scanning probe image processor (SPIP) software (*N* = 24). Two distinct populations were observed and peaks in the volume distribution are apparent at ∼250 and ∼400 nm^3^. (**F** and **G**) Time-lapse AFM images of complex of DNA frame with indicated *Il6* stem–loop RNAs and Regnase-1. Time 0 s is an arbitrary time during the AFM scanning. Scanning rate is 0.2 frames/s. See also Supplementary Figure S1 and Supplementary Movies S1 and S2.

To determine if Regnase-1 is recruited to its target mRNA following translation, we performed an RNA-immunoprecipitation (RIP) assay in HeLa cells transiently-expressing Flag-Regnase-1. As shown in Figure 1A and Supplementary Figure S1A, Regnase-1 interacts with its target mRNAs, including *IL6, PTGS2* and *NFKBIZ*, but not the non-target *NFKBIA*. Inhibition of translation by treatment with anisomycin did not decrease the RNA-Regnase-1 interaction, although anisomycin treatment completely blocked RMD (21). Noteworthy, we have previously shown that the interaction between Regnase-1 and target mRNA is UPF1-independent (21). These results indicate that translation and UPF1 are not necessary for the recruitment of Regnase-1 to its target stem–loop RNAs, and suggests that RMD requires an additional switch during translation in cells.

To investigate the switching mechanism of RMD, we tried to recapitulate RNA cleavage by Regnase-1 *in vitro*. To this end, we incubated recombinant Regnase-1 with target stem–loop RNAs corresponding to *Il6* 3′ UTR-(87–102) and *Nfkbiz* 3′ UTR-(103–119) (21). Unexpectedly, we found that *Il6* and *Nfkbiz* stem–loops were resistant to degradation by Regnase-1 at least 30 min of incubation in the cleavage buffer (Figure 1B and Supplementary Figure S1B). Since Regnase-1 prefers to cleave unstructured single-stranded RNA (ssRNA) in a sequence-independent manner *in vitro* (38), we hypothesized that the partially unwound stem–loop RNA can function as the substrate of Regnase-1. We thus performed *in vitro* cleavage assay with RNAs predicted to form only 2 nt stem–loop (stem mutant for *Il6* and *Nfkbiz* 3′ UTRs) and found that Regnase-1 cleaved the stem mutants (Figure 1B and Supplementary Figure S1A). A Regnase-1 mutant lacking RNase activity (D141N, DN) did not cleave the stem mutants (Supplementary Figure S1B). To directly visualize RMD at a single-molecule level, we performed time-lapse high-speed atomic force microscopy (AFM) analysis (33,34). AFM with DNA origami technology allows precise placement of target molecules in the designed nanostructures and enables molecules to be detected at the single-molecule level and we applied the AFM with DNA origami technology to visualizing and analyzing the RMD. Here, to examine the reaction, the *Il6* stem–loop and stem mutant RNAs were hybridized within a DNA origami frame (Figure 1C) and the RNA substrate attachment was observed by AFM (Figure 1D). We then incubated monomeric Regnase-1 (Figure 1E and Supplementary Figure S1C) with an *Il6* stem–loop RNA-DNA frame and found that *Il6* stem–loop RNA was not cleaved by Regnase-1 under the same condition as the *in vitro* cleavage assay, although Regnase-1 moved along the RNA and bound the *Il6* stem–loop RNA (Figure 1F and Supplementary Movie S1). In contrast, Regnase-1 rapidly degraded *Il6* stem mutant and dissociated from it (Figure 1G and Supplementary Movie S2). These results indicate that the stem–loop structure halts RMD, though it is necessary for Regnase-1 recognition.

### Stem-loop unwinding by UPF1 licenses RNA cleavage by Regnase-1

**Figure 2.**
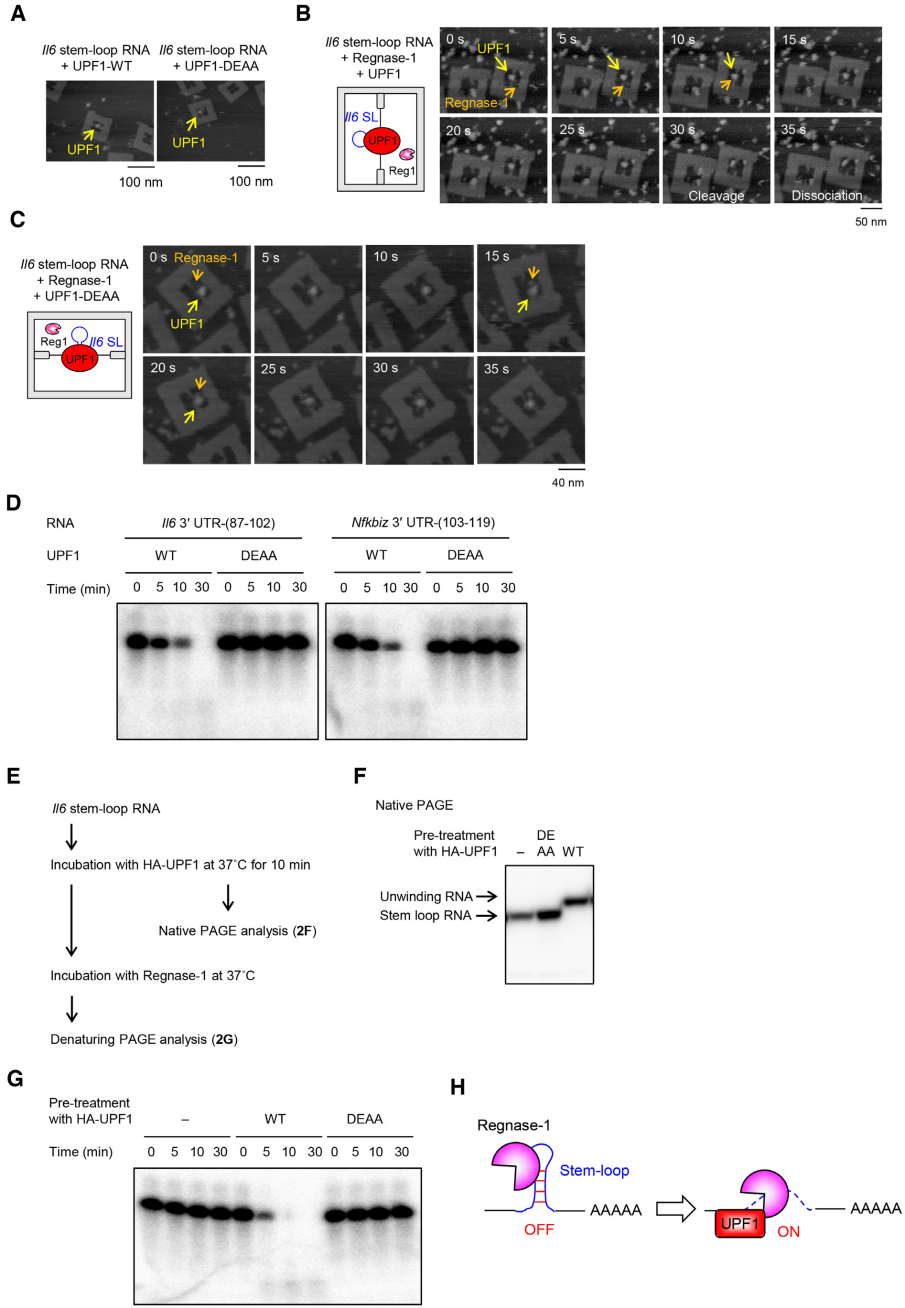
The unwinding of stem–loop by RNA helicase UPF1 induces RNA cleavage by Regnase-1. (**A**) AFM images of complex of DNA frame with *Il6* RNA and UPF1. (**B** and **C**) Time-lapse AFM images of complex of DNA frame with indicated *Il6* stem–loop RNA, Regnase-1 and UPF1. Time 0 s is an arbitrary time during the AFM scanning. Scanning rate is 0.2 frames/s. (**D**) Regnase-1 cleaved the stem–loop RNAs in the presence of UPF1 *in vitro*. Indicated 5′-^32^P-labeled RNAs were incubated with Regnase-1 and HA-UPF1 for indicated time, and analyzed by TBE–urea gel electrophoresis. (**E**) Flow chart depicting experiments where 5′-^32^P-labeled *Il6* stem–loop RNA was incubated with UPF1 at 37°C for 10 min, purified at 4°C and analyzed by native PAGE electrophoresis. To keep unwinding structure of stem–loop RNA by UPF1, RNA was immediately incubated and kept at 4°C after UPF1 treatment, and then incubated with Regnase-1. (**F**) Native PAGE analysis of *Il6* stem–loop RNA treated with UPF1. (**G**) Regnase-1 cleaved the *Il6* stem–loop RNA treated with UPF1 *in vitro*. The *Il6* stem–loop RNA treated with UPF1 were incubated with Regnase-1 for indicated time and analyzed by TBE–urea gel electrophoresis. (**H**) Schematic illustration showing that the unwinding of stem–loop by UPF1 induces RNA cleavage by Regnase-1. See also Supplementary Figure S1 and Supplementary Movies S3 and S4.

Since interaction between Regnase-1 and UPF1 during translation is essential for RMD (21), we hypothesized that the presence of UPF1 could be a key to recapitulating RMD *in vitro*. Therefore, we prepared wild-type (WT) and ATPase/helicase inactive mutant (D648A/E649A; DEAA) of UPF1 (Supplementary Figure S1D) (24). We next performed time-lapse high-speed AFM imaging in the presence of UPF1 and observed that a single molecule of UPF1 stably bound to the *Il6* stem–loop and did not cleave the RNA (Figure 2A). Interestingly, co-incubation of Regnase-1 and UPF1 on the *Il6* stem–loop-DNA frame led to rapid cleavage of the *Il6* stem–loop, and subsequent dissociation of Regnase-1 and UPF1 from the RNA (Figure 2B and Supplementary Movie S3). In contrast, Regnase-1 failed to cleave *Il6* stem–loop RNA in the presence of the DEAA mutant UPF1 (Figure 2C and Supplementary Movie S4). Furthermore, an *in vitro* RNA cleavage assay revealed that Regnase-1 degraded *Il6* and *Nfkbiz* stem–loops in the presence of WT, but not DEAA mutant UPF1 (Figure 2D and Supplementary Figure S1E), indicating that the UPF1 helicase activity is essential for the cleavage of stem–loop RNAs by Regnase-1. To investigate whether cleavage by Regnase-1 requires UPF1-mediated structural changes in the RNA, we initially pretreated *Il6* stem–loop RNA with UPF1 and checked for RNA structural changes (Figure 2E). We found that treatment with UPF1 indeed induced RNA unwinding (Figure 2F), and that the unwound RNA was cleaved by Regnase-1 even in the absence of UPF1 (Figure 2G and Supplementary Figure S1F). These results indicate that Regnase-1 is competent to act as a nuclease even without UPF1, and that the unwinding of the stem–loop by UPF1 licenses RNA cleavage by Regnase-1 (Figure 2H). The RNA structural change induced by UPF1 thus serves as a switch to execute RMD.

### Regnase-1 binding blocks UPF1 intramolecular auto-inhibition

**Figure 3.**
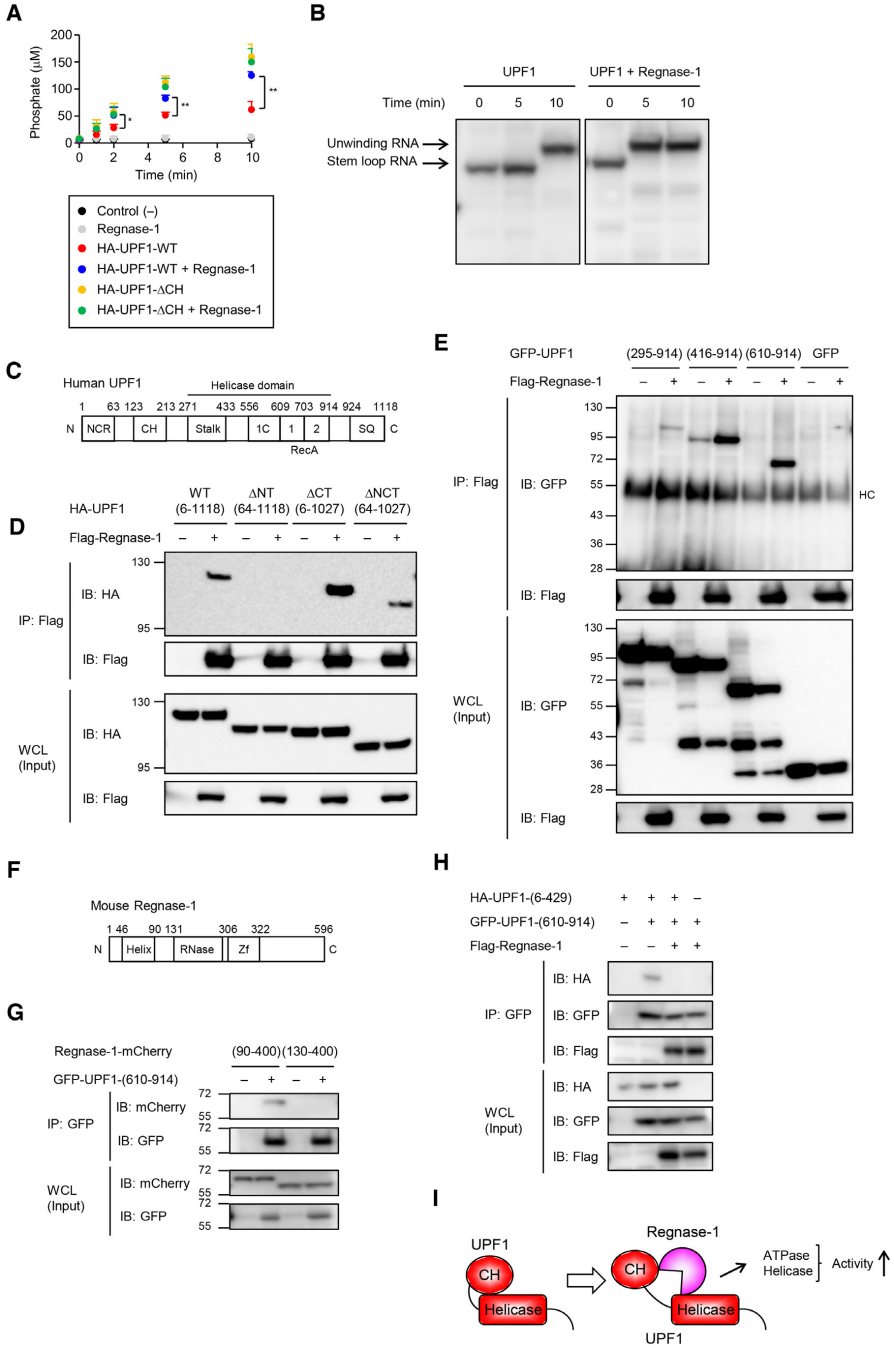
Regnase-1 promotes ATPase/helicase activity of UPF1 by inhibiting interaction between UPF1 RecA and CH domain. (**A**) ATPase assay of HA-UPF1 protein in the presence of Regnase-1. (**B**) Native PAGE analysis of *Il6* stem–loop RNA. The 5′-^32^P-labeled RNA was incubated with UPF1 and Regnase-1, purified at 4°C and then analyzed by native polyacrylamide gel electrophoresis. (**C**) Schematic representation of human UPF1. NCR, N-terminal conserved region; CH, cysteine/histidine-rich domain; RecA, RecA-like domain; SQ, serine/glutamine motif. (**D**) Western blot analysis of HA-UPF1 and indicated UPF1 mutants co-immunoprecipitated with Flag-Regnase-1 transiently-expressed in HeLa cells. (**E**) Immunoblot analysis of SBP-GFP-UPF1 and its deletion mutants co-immunoprecipitated with Flag-Regnase-1 transiently-expressed in HeLa cells. (F) Schematic representation of mouse Regnase-1. (**G**) Western blot analysis of indicated Regnase-1-mCherry deletion mutants co-immunoprecipitated with GFP-UPF1-(610–914) or GFP-UPF1-(6–429)-T28E transiently-expressed in HeLa cells. (**H**) Immunoblot analysis of HA-UPF1-(6–429) and Flag-Regnase-1 co-immunoprecipitated with GFP-UPF1-(610–914) transiently-expressed in HeLa cells. (**I**) Schematic illustration showing that Regnase-1 promotes ATPase/helicase activity of UPF1 by inhibiting interaction between UPF1 RecA and CH domain. See also Supplementary Figure S1.

We next addressed the question of how the RNA structural change is regulated by UPF1 in response to translation termination. Interestingly, the presence of Regnase-1 significantly enhanced the ATPase activity of UPF1 *in vitro* (Figure 3A). UPF1 DEAA mutant showed no ATPase activity in the presence of Regnase-1 (Supplementary Figure S1G). Moreover, measurement the helicase activity of UPF1 by native gel analysis of *Il6* stem–loop RNA revealed that addition of Regnase-1 accelerated the unwinding of RNA by UPF1 (Figure 3B). These results indicate that the interaction between UPF1 and Regnase-1 promotes the ATPase and helicase activities of UPF1.

We next investigated the mechanism of Regnase-1-mediated control of UPF1 by identifying regions responsible for their interaction. UPF1 harbors an N-terminal regulatory region followed by a cysteine-histidine-rich domain (CH-domain), a RecA helicase domain and a C-terminal regulatory region (Figure 3C). The CH domain functions to suppresses the ATPase/helicase activity via intramolecular interactions with the RecA domain (39). Consistent with the previous study, the ATPase activity of a truncated mutant of UPF1 (ΔCH, aa 295–914) was higher than that of WT UPF1 (Supplementary Figure S1D). Regnase-1 had no effect on ATPase activity of UPF1-ΔCH mutant (Figure 3A). Co-immunoprecipitation using UPF1 lacking the N-terminal (ΔN), C-terminal (ΔC) or both the N- and C-terminal regions (ΔNCT) revealed that full-length and ΔNCT, but not ΔN, UPF1 associated with Regnase-1 (Figure 3D), suggesting that UPF1 harbors two distinct Regnase-1-interaction regions in the N-terminus and the central parts. Regnase-1 interacted with ΔCT UPF1 more robustly than WT and ΔNCT UPF1 (Figure 3D), suggesting that the C-terminal region of UPF1 functions as inhibitory domain for association with Regnase-1. Noteworthy, we have previously shown that the interaction between Regnase-1 and UPF1 was not affected by the treatment of cell lysates with an RNase Benzonase, indicating that Regnase-1 and UPF1 directly interact at the protein levels (21). Further co-immunoprecipitation assays identified that the RecA domain (610–914) in the central region of UPF1 was responsible for the association with a structurally disordered region (90–130) of Regnase-1 (Figure 3E–G). Interestingly, the intramolecular interaction between the N-terminal half of UPF1 harboring the CH domain (6–429) and RecA domain (610–914) was inhibited by the co-expression of Regnase-1 (Figure 3H). This finding suggests that the binding of Regnase-1 to UPF1 RecA domain inhibits the intramolecular interaction between the RecA and CH domains of UPF1, allowing the activation of the UPF1 ATPase/helicase activity to trigger RMD (Figure 3I).

### UPF1 T28 phosphorylation stabilizes interaction between UPF1 and Regnase-1

**Figure 4.**
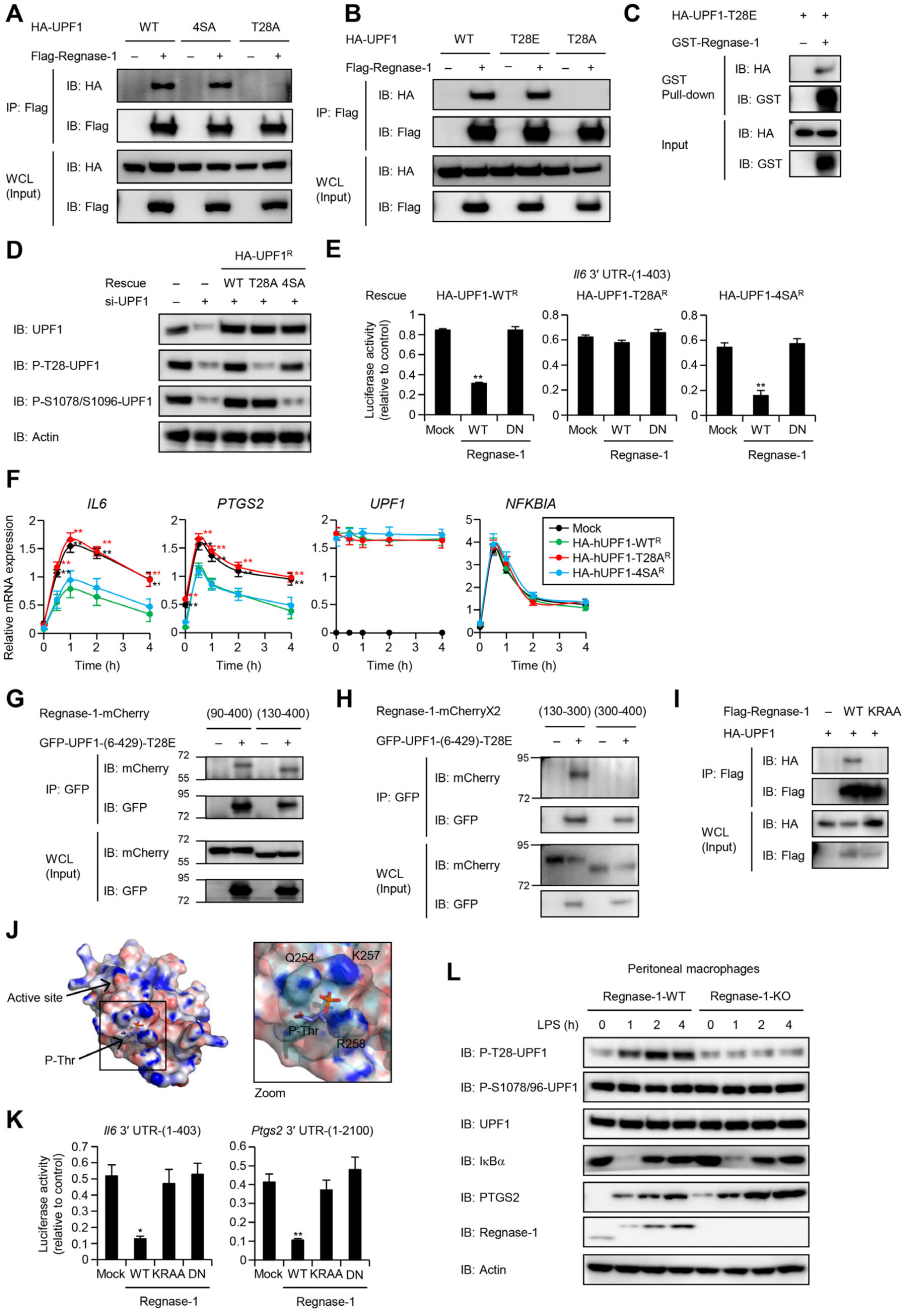
Phosphorylation of UPF1 at T28 is required for RMD. (**A** and **B**) Western blot analysis of HA-UPF1 and indicated UPF1 mutants co-immunoprecipitated with Flag-Regnase-1 transiently-expressed in HeLa cells. (**C**) Western blot of GST pull-down assay showing the direct association of Regnase-1 with UPF1. (**D**) Immunoblot analysis of UPF1 in HeLa cells transfected with siRNA specific for UPF1 and reconstituted with siRNA-resistant HA-UPF1^R^-WT or indicated its mutants. (**E**) Luciferase activity of HeLa cells transfected with siRNA specific for UPF1 and reconstituted with siRNA-resistant WT UPF1 or indicated its mutants and then transfected with indicated luciferase reporter plasmids, together with control plasmid (Mock) or Regnase-1 expression plasmids. (**F**) RNA expression levels in HeLa cells transfected with siRNA specific for UPF1, reconstituted with siRNA-resistant WT UPF1 or indicated its mutants. The cells were stimulated with IL-1β (10 ng/ml). (**G** and **H**) Western blot analysis of indicated Regnase-1-mCherry deletion mutants co-immunoprecipitated with GFP-UPF1-(610–914) or GFP-UPF1-(6–429)-T28E transiently-expressed in HeLa cells. (**I**) Immunoblot analysis of HA-UPF1 co-immunoprecipitated with Flag-Regnase-1 and its K257A/R258A (KRAA) mutant transiently-expressed in HeLa cells. (**J**) Structural model of phosphothreonine (P-Thr) docked to homology model of mouse Regnase-1 RNase domain (134–296). (**K**) Luciferase activity of HeLa transfected with indicated luciferase reporter plasmids, together with control plasmid (Mock) or indicated expression plasmids. Unless otherwise indicated, data are mean ± SD (*n* = 3). (**L**) Immunoblot analysis of P-T28-UPF1 and P-S1078/S1096-UPF1 in peritoneal macrophages from *Regnase-1^+/+^* (WT) and *Regnase-1^–/–^* (KO) mice. Macrophages were stimulated with LPS (100 ng/ml). See also Supplementary Figures S1 and S2.

In addition to the RecA domain, the N-terminal region of UPF1 is essential for binding with Regnase-1. In NMD, UPF1 N- and C-terminal regions (T28, S1078, S1096 and S1116) are phosphorylated and bind with SMG6 and SMG5-7, respectively (7,28,29,40). Interestingly, a single point mutation of T28 to alanine (T28A), but not to glutamate (T28E) or mutations at the C-terminal phosphorylation sites (4SA), abrogated the association of Regnase-1 with UPF1 (Figure 4A and B and Supplementary Figure S1H). Indeed, treatment of cell lysates with λ protein phosphatase reduced the association between Regnase-1 and UPF1 (Supplementary Figure S1I). Reciprocally, a hyperphosphorylated UPF1 variant (G495R/G497E) (Supplementary Figure S2A) (41,42) showed increased association with Regnase-1 (Supplementary Figure S2B). To examine direct Regnase-1-UPF1 interaction *in vitro*, we performed GST pull-down assay with Regnase-1 and UPF1-T28E mutant and found that Regnase-1 directly associated with UPF1-T28E (Figure 4C). These results indicate that UPF1 T28 phosphorylation is critical for stable association with Regnase-1. Consistently, reconstitution of UPF1 knockdown HeLa cells with WT and 4SA, but not T28A, UPF1 rescued Regnase-1-mediated suppression of a luciferase reporter harboring *Il6* 3′ UTR (17,21) (Figure 4D and E). Similarly, suppression of IL-1β-mediated expression of *IL6* and *PTGS2* required T28, but not 4S, phosphorylation of UPF1 (Figure 4F). In contrast, both T28A and 4SA UPF1 mutants failed to suppress NMD target genes such as *SMG5* and *GAS5* (Supplementary Figure S2C). Collectively, these results demonstrate that T28, but not C-terminal, phosphorylation of UPF1 is essential for RMD.

We next asked how Regnase-1 recognizes the phosphorylated UPF1 N-terminus. We found that the RNase domain (130–300) of Regnase-1 interacted with the T28-phosphorylated (negatively charged) N-terminal half (6–429) of UPF1 (Figure 4G and H). By constructing a structural model of the Regnase-1 RNase domain (134–296), we identified four regions (regions 1–4) on the surface of the Regnase-1 RNase domain that could potentially recognize the phospho moiety via electrostatic interactions (Supplementary Figure S2D and E). Among these, mutations of region 4 (K257A/R258A; KRAA), but not regions 1–3, abrogated the interaction with WT and phosphomimetic T28E UPF1 (Figure 4I and Supplementary Figure S2F). Indeed, a structural model of phosphothreonine (P-Thr) docked to Regnase-1 RNase domain showed that K257R258 and glutamine 254 (Q254) form a positively charged P-Thr binding pocket (Figure 4J). Although K257 and R258 in Regnase-1 are dispensable for its RNase activity *in vitro* (43), a Regnase-1-KRAA mutant failed to suppress *Il6* and *Ptgs2* 3′ UTRs (Figure 4K). These results suggest that Regnase-1 requires N-terminal UPF1 phosphorylation to form a stable association with UPF1, which, in turn, increases its helicase activity.

### Regnase-1 induces T28 phosphorylation of UPF1 after association with the RecA domain

The results above indicate that UPF1 and Regnase-1 interact in two distinct modes: UPF1 RecA with Regnase-1 linker regions, and UPF1 P-T28 with Regnase-1 RNase domain via K257/R258. Given that both interactions are required for RMD in cells, we investigated their hierarchy by focusing on the T28 phosphorylation of UPF1. First, we discovered that the UPF1 T28 phosphorylation was induced in macrophages stimulated with LPS (Figure 4L). This stimulus upregulates the expression of Regnase-1 target mRNAs such as *IL6* and *NFKBIZ*. We previously reported that Regnase-1 protein is phosphorylated and re-expressed in macrophages in response to LPS stimulation (17,19). Regnase-1 phosphorylation was detected as the mobility shift of the Regnase-1 bands (Figure 4L). Interestingly, overexpression of Regnase-1 target mRNA also augmented P-T28-UPF1, whereas C-terminal phosphorylation of UPF1 was unchanged (Supplementary Figure S2G). Surprisingly, UPF1 T28 phosphorylation was not induced in Regnase-1-deficient macrophages (Figure 4L), indicating that Regnase-1 is required for the phosphorylation of UPF1 T28 upon inflammatory stimuli.

We next investigated how Regnase-1 induces UPF1 T28 phosphorylation by using HEK293T cells in which Regnase-1 is rarely expressed. In this cell line, the expression of mRNAs harboring *Il6* 3′ UTR together with WT Regnase-1 induced UPF1 T28 phosphorylation (Supplementary Figure S2H). Whereas the mutation of K257/R258 in the Regnase-1 RNase domain (KKAA) did not affect induced phosphorylation of UPF1, lack of the linker (90–130) region of Regnase-1 abrogated the phosphorylation. These results suggest that the (90–130) region of Regnase-1 initially associates with the UPF1 RecA helicase domain during translation, which allows phosphorylation of UPF1 at T28, which is subsequently recognized by the RNase domain of Regnase-1 to mediate stable interaction between UPF1 and Regnase-1.

### SMG1 regulates RMD through phosphorylation of UPF1

**Figure 5.**
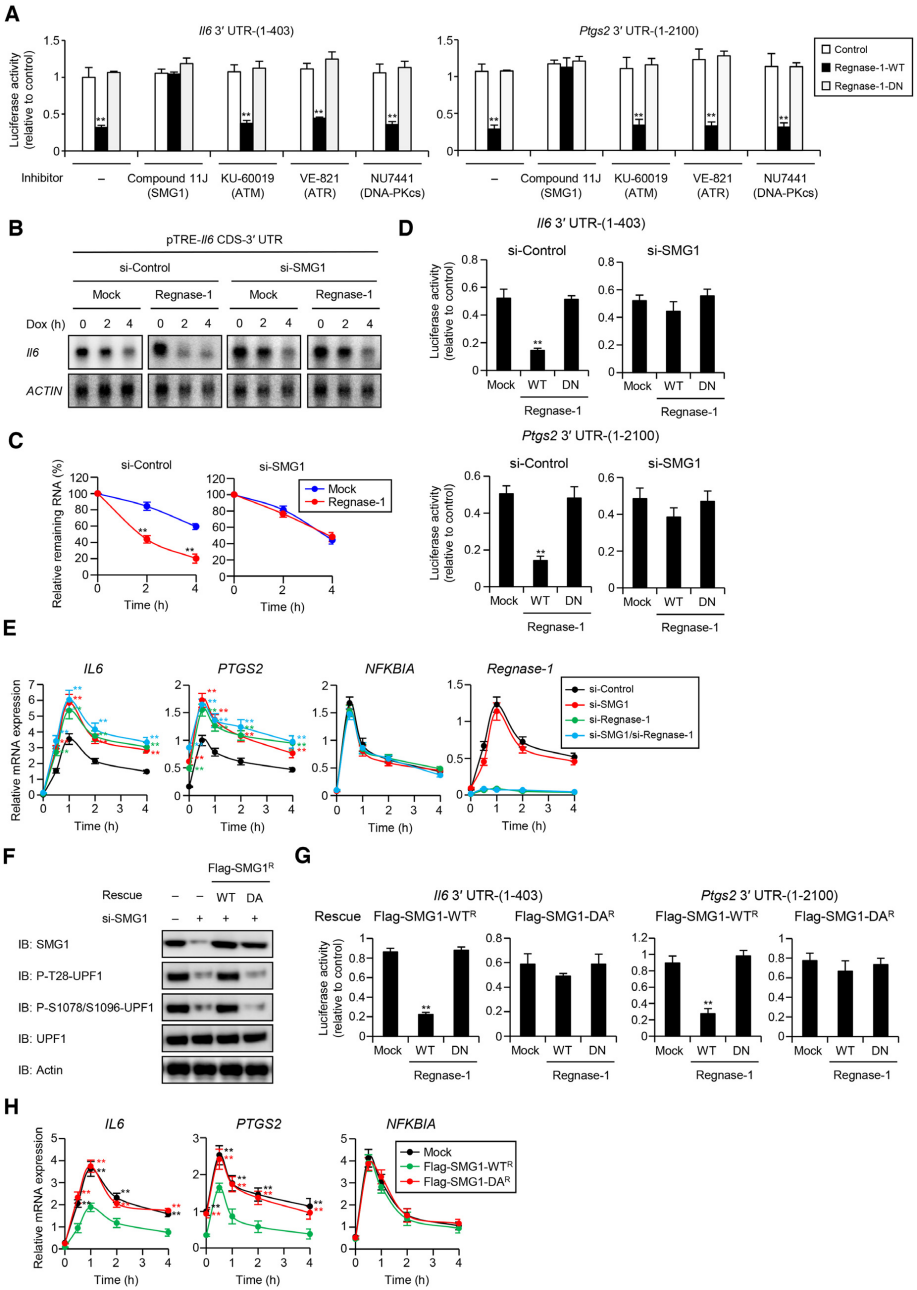
SMG1 regulates RMD through the direct phosphorylation of UPF1. (**A**) Luciferase activity of HeLa cells treated with indicated inhibitors, including Compound 11J (SMG1 inhibitor, 0.3 μM), KU-60019 (ATM inhibitor, 1 μM), VE-821 (ATR inhibitor, 1 μM) and NU7441 (DNA-PK inhibitor, 1 μM), and transfected with indicated luciferase reporter plasmids, together with control plasmid or indicated expression plasmids. (**B**) HEK293 Tet-off cells were transfected with siRNA specific for SMG1 and cotransfected with pTREtight-*Il6* CDS-3′ UTR together with the Regnase-1 expression plasmid or control (Mock) plasmid. Total RNAs were prepared after Dox (1 μg/ml) treatment, and *Il6* and *β-Actin* mRNA levels were determined by Northern blot analysis. (**C**) Quantification of the autoradiographs in (B), presented as the ratio of *Il6* to *β-Actin* (*n* = 3). (**D**) Luciferase activity of HeLa cells transfected with indicated siRNA and luciferase reporter plasmids, together with control plasmid (Mock) or Regnase-1 expression plasmids. (E) RNA expression levels in HeLa cells transfected with indicated siRNA and stimulated with IL-1β (10 ng/ml). (**F**) Western blot analysis of SMG1 in HeLa cells transfected with siRNA specific for SMG1 and reconstituted with siRNA-resistant Flag-SMG ^R^-WT or Flag-SMG1^R^-D2331A (DA), which is a kinase dead mutant. (**G**) Luciferase activity of HeLa cells transfected with siRNA specific for SMG1 and reconstituted with siRNA-resistant WT or DA mutant of SMG1 and then transfected with indicated luciferase reporter plasmids, together with control plasmid (Mock) or Regnase-1 expression plasmids. (H) RNA expression levels in HeLa cells transfected with siRNA specific for SMG1, reconstituted with siRNA-resistant WT or DA mutant of SMG1 and then stimulated with IL-1β (10 ng/ml). See also Supplementary Figure S3.

UPF1 is a shared substrate of phosphatidylinositol 3-kinase (PI3K)-related protein kinases (PIKKs), including SMG1, ataxia-telangiectasia mutated (ATM), ATM- and Rad3-related (ATR) and DNA-dependent protein kinase catalytic subunit (DNA-PKcs) kinases (44). Therefore, we explored kinase(s) essential for RMD through phosphorylation of UPF1 by treatment with their specific inhibitors. Interestingly, inhibition of SMG1, but not ATM, ATR or DNA-PKcs, abrogated Regnase-1-mediated suppression of a luciferase reporter harboring *Il6* and *Ptgs2* 3′ UTR (Figure 5A), suggesting that SMG1 is the sole kinase essential for Regnase-1 function. Furthermore, knockdown of SMG1 in HeLa cells inhibited T28-UPF1 phosphorylation and the association between Regnase-1 and UPF1 (Supplementary Figure S3A and B). When we knocked down SMG1 in HEK293 Tet-off cells, overexpression of Regnase-1 failed to destabilize *Il6* mRNA (Figure 5B and C). Furthermore, Regnase-1 overexpression no longer suppressed the luciferase reporter harboring *Il6* and *Ptgs2* 3′ UTRs in SMG1 knockdown cells (Figure 5D). Moreover, knockdown of SMG1 or Regnase-1 in HeLa cells resulted in increased expression of endogenous *IL6* and *PTGS2*, but not *NFKBIA*, in response to IL-1β stimulation (Figure 5E). Notably, knockdown of both Regnase-1 and SMG1 did not lead to additional increase in gene expression, suggesting that Regnase-1 and SMG1 suppress gene expression via the same pathway. Knockdown of Regnase-1, but not SMG1, had no effect of expression of classical NMD substrates, *GAS5* and *SMG5*, suggesting that Regnase-1 has no effect on NMD (Supplementary Figure S3C). Reconstitution of SMG1 knockdown cells with WT, but not kinase inactive mutant (D2331A, DA) SMG1 (28,45), rescued Regnase-1-mediated suppression of *Il6* and *Ptgs2* 3′ UTRs (Figure 5F and G) as well as inhibition of *IL6* and *PTGS2* in IL-1β-stimulated HeLa cells (Figure 5H). Reconstitution with WT SMG1, but not DA mutant, rescued NMD (Supplementary Figure S3D). These results indicate that SMG1 is an essential kinase for the regulation of RMD through phosphorylation of UPF1.

### Regnase-1 suppresses inflammatory mRNAs following a pioneer round of translation

We next sought to identify the biological significance of the unique translation-dependent RMD molecular switch. We first focused on the character of mRNAs degraded by Regnase-1 during translation. Newly synthesized mRNAs bound by the nuclear cap-binding protein complex CBP80/20 (CBC) undergo a pioneer round of translation, and CBC-bound mRNPs are remodeled to the cytoplasmic cap-binding protein eIF4E for steady state translation (46,47). NMD targets CBP80-bound mRNAs in addition to eIF4E-associated mRNAs (48,49). Interestingly, co-immunoprecipitation experiments revealed that catalytically active Regnase-1 bound to CBP80-associated, but not to eIF4E-bound, mRNAs in HeLa cells (Supplementary Figure S3E). In contrast, Regnase-1-DN mutant co-precipitated with both CBP80 and eIF4E, suggesting that Regnase-1 degrades target mRNAs before they can be bound by eIF4E mRNAs. This interaction was lost by treatment with Benzonase before immunoprecipitation, indicating that they are RNA-mediated interactions, but not direct protein-protein interactions.

**Figure 6.**
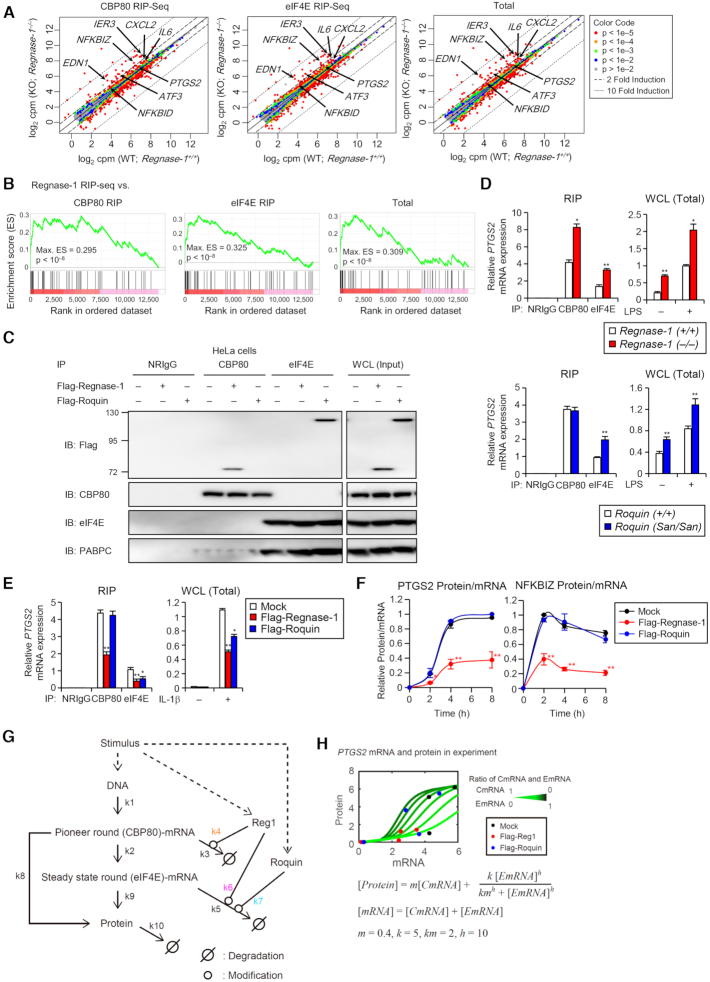
Regnase-1 binds and suppresses inflammatory mRNAs undergoing pioneer rounds of translation. (**A**) Scatter plot of expression changes of CBP80- and eIF4E-associated mRNAs upon IL-1β stimulation (10 ng/ml, 2 h) in Regnase-1-KO HeLa cells. CBP80- and eIF4E-associated mRNAs were immunoprecipitated and analyzed by mRNA-sequence in biological duplicate. Regnase-1 target mRNAs defined from Regnase-1 RIP-Seq (*P* < 0.001) were indicated. (**B**) GSEA of overlap between 68 genes enriched in Regnase-1 RIP-seq (*P* < 0.001) and genes sorted by CBP80 or eIF4E RIP-seq enrichments in Regnase-1-KO cells. The ES plots were shown with genes ranked according to their CBP80 or eIF4E RIP-seq enrichments *P* value. (**C**) Western blot analysis of Flag-Regnase-1 and Flag-Roquin-1 co-immunoprecipitated with CBP80 or eIF4E in HeLa cells. (**D**) RNA expression levels of CBP80- and eIF4E-associated mRNAs in MEFs stimulated with LPS (100 ng/ml) for 2 h. (**E**) RNA expression levels of CBP80- and eIF4E-associated mRNAs in HeLa cells transfected with Flag-Regnase-1 or Flag-Roquin expression plasmid and stimulated with IL-1β (10 ng/ml) for 2 h. (**F**) The protein/mRNA ratio of PTGS2 and NFKBIZ in HeLa cells transfected with the control (Mock), Flag-Reg1 or Flag-Roquin expression plasmid and stimulated with IL-1β (10 ng/ml). RNA and protein expression profiles of PTGS2 and NFKBIZ are shown in Figures S6A and S6B. The data were normalized to maximum values. (**G**) Diagram of transcription-translation process mediated by Regnase-1 (Reg1) and Roquin. CBP80-bound mRNA and eIF4E-bound mRNA were described as CBP80-mRNA and eIF4E-mRNA, respectively. Dashed arrow indicates stimulus-induced activation. Solid arrow indicates reactions of transcription, translation and degradation. (H) Fitting of the mathematical models to the experimental data. Circles indicate experimental values of PTGS2 mRNA and protein in each condition. Green lines indicate simulation results in different numerical conditions where stoichiometric ratios of CBP80-bound mRNA (CmRNA) and eIF4E-bound mRNA (EmRNA) are varied between 0 to 1. See also Supplementary Figure S3–S6.

We then investigated whether Regnase-1 has the potential to destabilize mRNAs at the pioneer round of translation. We performed an RIP assay with CBP80 or eIF4E antibodies in *Regnase-1*-deficient (*Regnase-1^–/–^*; KO) HeLa cells (Supplementary Figure S3F and G). Regnase-1 deficiency caused the increase of CBP80-bound mRNAs, including the target *IL6, PTGS2* and *NFKBIZ*, but not the non-target *NFKBIA*. Regnase-1 targets also increased in eIF4E-associated mRNAs in *Regnase-1^–/–^* cells, since CBP80 is replaced with eIF4E. To examine the impact of Regnase-1 on CBP80-bound mRNAs comprehensively, we performed RNA-immunoprecipitation sequencing (RIP-seq) for CBP80- or eIF4E-associated mRNAs in *Regnase-1^–/–^* HeLa cells. Regnase-1-targeted mRNAs, including *IL6, PTGS2, NFKBIZ* and *NFKBID*, were enriched, even in CBP80-bound mRNAs from *Regnase-1^–/–^* HeLa cells, compared with control (Figure 6A). Most genes were plotted on the diagonal line in the scatter plot of fold-changes between Regnase-1-WT and -KO in CBP80-RIP against eIF4E-RIP and the difference between Regnase-1-WT and -KO in the eIF4E-RIP was almost the same as in the CBP80-RIP (Supplementary Figure S3H), suggesting that Regnase-1 regulates CBP80-mRNAs. We have previously identified 68 Regnase-1-associated mRNAs by RIP-seq analysis (21). Gene set enrichment analysis (GSEA) revealed that the 68 Regnase-1-interacting mRNAs were significantly enriched in the CBP80-bound mRNAs in *Regnase-1^–/–^* cells with high enrichment scores (ES) (Max. ES = 0.295, *P* < 10^−8^; Figure 6B). These results are consistent with a scenario in which Regnase-1 deficiency upregulates target mRNAs at the pioneer round of translation.

We next explored whether the regulation of mRNAs in the pioneer round of translation is common to RNA-binding proteins controlling immune responses. We have reported that Regnase-1 and Roquin recognize common stem–loop structures (21), though Roquin-mediated degradation of inflammatory mRNAs is not dependent on UPF1. In contrast to Regnase-1, both overexpressed and endogenous Roquin bound eIF4E-associated, but not CBP80-associated, mRNAs (Figure 6C and Supplementary Figure S3E). Consistently, the *Roquin-1^San/San^* mouse embryonic fibroblast (MEFs), harboring a M199R hypomorphic mutation in *Roquin-1* alleles (50,51), showed increased expression of eIF4E-bound, but not CBP80-bound, target mRNAs compared with WT cells upon LPS stimulation (Figure 6D and Supplementary Figure S4A). On the other hand, *Regnase-1^–/–^* MEFs exhibited even an increase in CBP80-bound mRNAs. Moreover, Regnase-1 deficiency increased the mRNA half-lives (*t*_1/2_) of CBP80- as well as eIF4E-bound mRNAs encoding *Ptgs2* and *Nfkbiz* in MEFs, although *Roquin-1^San/San^* MEFs showed the increased t_1/2_ of eIF4E-bound mRNAs (Supplementary Figure S4B). Reciprocally, overexpressed Regnase-1 decreased both CBP80- and eIF4E-bound mRNAs encoding *PTGS2* and *NFKBIZ* in HeLa cells upon IL-1β stimulation; whereas, overexpression of Roquin destabilized eIF4E-bound mRNAs alone (Figure 6E and Supplementary Figure S5A). Furthermore, overexpression of Regnase-1 reduced the *t*_1/2_ of CBP80- as well as eIF4E-bound mRNAs, although overexpression of Roquin decreased the *t*_1/2_ of eIF4E-bound mRNAs alone (Supplementary Figure S5B). Notably, knockdown of SMG1 also increased CBP80- as well as eIF4E-bound Regnase-1-target mRNAs (Supplementary Figure S5C). Taken together, the SMG1-Regnase-1 pathway potently destabilizes mRNAs undergoing pioneer rounds of translation, whereas Roquin regulates mRNAs that have undergone multiple rounds of translation.

### Modeling inflammatory gene expression from pioneer and steady state rounds of translation

We next addressed the question of how RMD following pioneer rounds of translation contribute to the regulation of inflammation. By experimental analysis on the effects of Regnase-1 and Roquin in the expression of target mRNAs and proteins, we found that Regnase-1 is potent to suppress the protein/mRNA ratio compared to control cells (Figure 6F and Supplementary Figure S6A and B). In contrast, Roquin expression affected mRNA and protein expression to a similar extent, though both Regnase-1 and Roquin-based degradation exhibit similar kinetics.

These experimental findings prompted us to decipher the dynamics of post-transcriptional regulation systems of inflammation by Regnase-1 and Roquin using a mathematical model. Our model includes, along with the translated proteins, two states for the mRNAs: those undergoing a pioneer round (CBP80-bound) and those undergoing steady state rounds (eIF4E-bound) of translation (Figure 6G). Based on the experimental results, we assumed that Regnase-1 induces degradation of both the CBP80- and eIF4E-bound fractions of mRNAs, and Roquin only degrades the eIF4E-bound mRNA fraction. Because eIF4E-mRNAs mediate steady-state translation and it is considered that steady-state translation synthesizes much protein in polysome state, we assumed that the input-output relationship between mRNA and protein is nonlinear in eIF4E-mRNAs. On the other hand, CBP80-mRNAs mediate pioneer round of translation and it is considered that pioneer round of translation produces less protein than steady-state translation. Thus, we hypothesize that the relationship between mRNA and protein is linear in CBP80-mRNAs. Indeed, we confirmed, by fitting an assumed equation (Figure 6H, green line), that the input-output relationship between mRNA and protein in the experiment is nonlinear in eIF4E-bound mRNAs, probably representing that steady-state translation synthesizes much protein in polysome state. This effect was incorporated into the model by representing eIF4E-bound (*E*) mRNA translation by a Hill equation, while CBP80-bound mRNA translation was represented as a linear model (Figure 6H, Method). Simulation using the model qualitatively recapitulated dynamics of protein/mRNA in PTGS2 and NFKBIZ (Supplementary Figure S6C and S6D, for experimental data 6F, S5A, S6A, Supplementary Tables S1 and S2), indicating that the model essentially explains the time-course kinetics of the translation process and therefore can generate predictions of underlying mechanisms. To compare influences of Regnase-1 and Roquin on the dynamics of the synthesized protein, we checked the translation rates of protein from CBP80- and eIF4E-bound mRNA in the simulation results. Interestingly, the translation rate of eIF4E-bound mRNA in Flag-Regnase-1-overexpressed cells was significantly lower than in the WT and Flag-Roquin expressing cells (Supplementary Figure S6E, circles). Given that Regnase-1 suppresses CBP80-bound mRNA, which further affects eIF4E-bound mRNA, the amount of eIF4E-mRNA in Flag-Regnase-1 cells was not beyond a threshold (km) value; therefore, it could not efficiently translate proteins (Supplementary Figure S6E, red circle, Supplementary Figure S6F). On the other hand, translation rates of CBP80-mRNA were comparable in Regnase-1- and Roquin-expressing cells. To investigate the reason why the difference in the translation rates of eIF4E-bound mRNA occurred, we performed sensitivity analysis which examines how perturbations to the Regnase-1- (k4, k6) and Roquin-induced degradation (k7) in the model affect the eIF4E-mRNA. This analysis showed that Regnase-1-induced degradation (k4) in *PTGS2* and *NFKBIZ* yield the greatest change in eIF4E-mRNA, even if Roquin is overexpressed (Supplementary Figure S6G) and the effect is transmitted to the protein level (Supplementary Figure S6H). Taken together, our mathematical model reveals that the effects of Regnase-1 and Roquin can best be recapitulated by considering pioneer and steady state rounds of translation separately.

### Control of DC maturation and proinflammatory gene expression by SMG1 inhibition

**Figure 7.**
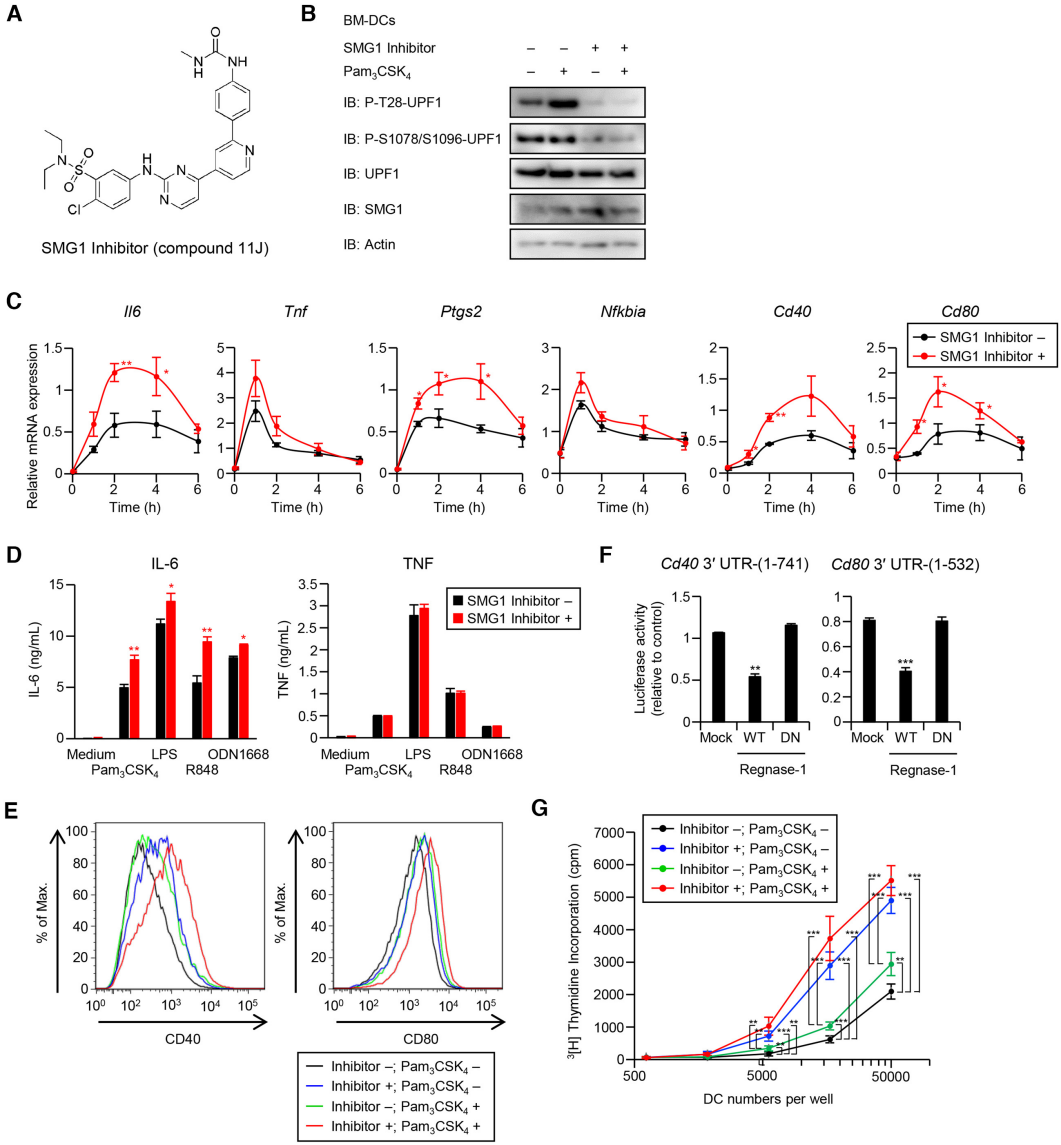
Control of DC maturation by manipulating SMG1 activity. (**A**) Chemical structure of SMG1 inhibitor. (**B**) Immunoblot analysis of P-T28-UPF1 and P-S1078/S1096-UPF1 in BMDCs cultured with Pam_3_CSk_4_ (1 ng/ml) and/or SMG1 inhibitor (0.3 μM) for 2 h. (**C**) RNA expression levels in BMDCs. The BMDCs from C57BL/6J mice were treated with SMG1 inhibitor (0.3 μM) and stimulated with Pam_3_CSK_4_ (1 ng/ml). (**D**) BMDCs from C57BL/6J mice were treated with SMG1 inhibitor (0.3 μM) and stimulated with Pam_3_CSK_4_ (10 ng/ml), LPS (100 ng/ml), R848 (100 nM) or CpG DNA (ODN1668, 100 nM) for 24 h. Production of IL-6 and TNF in the culture supernatant were measured by ELISA. (**E**) Expression levels of CD40 and CD80 on BMDCs. The BMDCs from C57BL/6J mice were cultured with Pam_3_CSK_4_ (1 ng/ml) and/or inhibitor (0.3 μM SMG1) for 48 h and expression of CD40 and CD80 on BMDCs were analyzed by Flow cytometry. (**F**) Luciferase activity of HEK293T cells transfected with indicated luciferase reporter plasmids, together with control plasmid (Mock) or Regnase-1 expression plasmids. (**G**) Allogeneic mixed lymphocyte reaction of splenic CD4^+^ T cells from C57BL/6J mice with BMDCs from BALB/c mice cultured with SMG1 inhibitor (0.3 μM) and Pam_3_CSK_4_ (1 ng/ml) for 48 h. See also Supplementary Figure S7.

The requirement of SMG1 in RMD prompted us to examine the possibility of immune responses being manipulated by controlling SMG1 activity. When we treated bone marrow–derived DCs (BMDCs) with a SMG1 inhibitor (referred to as ‘Compound 11J’ in (27)) (Figure 7A), the phosphorylation of UPF1 at T28 was profoundly suppressed in unstimulated or Pam_3_CSK_4_ (TLR2 ligand)-treated cells (Figure 7B). Treatment with the SMG1 inhibitor in WT BMDCs resulted in increased expression of *Il6* and *Ptgs2*, but not *Tnf* and *Nfkbia*, in response to Pam3CSK4 stimulation (Figure 7C). Lack of Regnase-1 increased inflammatory gene expression (Supplementary Figure S7A and S7B) and the SMG1 inhibitor had no effect on their expression in *Regnase-1^–/–^*BMDCs (Supplementary Figure S7C), indicating that SMG1 regulates cytokine expression in a Regnase-1-dependent manner. In addition to proinflammatory cytokines, the expression levels of co-stimulatory molecules, *Cd40* and *Cd80*, was enhanced by the treatment with the SMG1 inhibitor in BMDCs (Figure 7C). Production of IL-6, but not TNF, was increased by treatment with the SMG1 inhibitor in response to all TLR ligands, Pam_3_CSK_4_ (TLR2), LPS (TLR4), R848 (TLR7) and CpG-DNA (TLR9), in BMDCs (Figure 7D), indicating that SMG1 regulates cytokine expression at the post-transcriptional level through the Regnase-1 pathway in innate immune cells.

Next, we investigated the effect of SMG1 inhibition on the maturation of DCs. Treatment with the SMG1 inhibitor augmented the surface expression of CD40 and CD80 with or without stimulation with a TLR2 ligand (Figure 7E). As expected, expression levels of CD40 and CD80 increased in *Regnase-1^–/–^* BMDCs (Figure S7D) and *Cd40* and *Cd80* mRNA expressions were also upregulated in *Regnase-1^–/–^* BMDCs compared with wild-type controls (Supplementary Figure S7B). Moreover, Regnase-1 overexpression suppressed the luciferase reporter construct harboring *Cd40* and *Cd80* 3′ UTRs in an RNase activity-dependent manner (Figure 7F). Consistent with augmented expression of co-stimulatory molecules, allogeneic mixed lymphocyte reaction (MLR) assays of splenic CD4^+^ T cells with BMDCs revealed that treatment with a SMG1 inhibitor alone increased DC-induced T cell stimulatory activity, which was further enhanced by co-stimulation with Pam_3_CSK_4_ (Figure 7G). Collectivity, these data demonstrate that DC maturation can be precisely manipulated by controlling SMG1 kinase activity by using an inhibitor via Regnase-1-mediated inflammatory mRNA degradation.

## DISCUSSION

**Figure 8.**
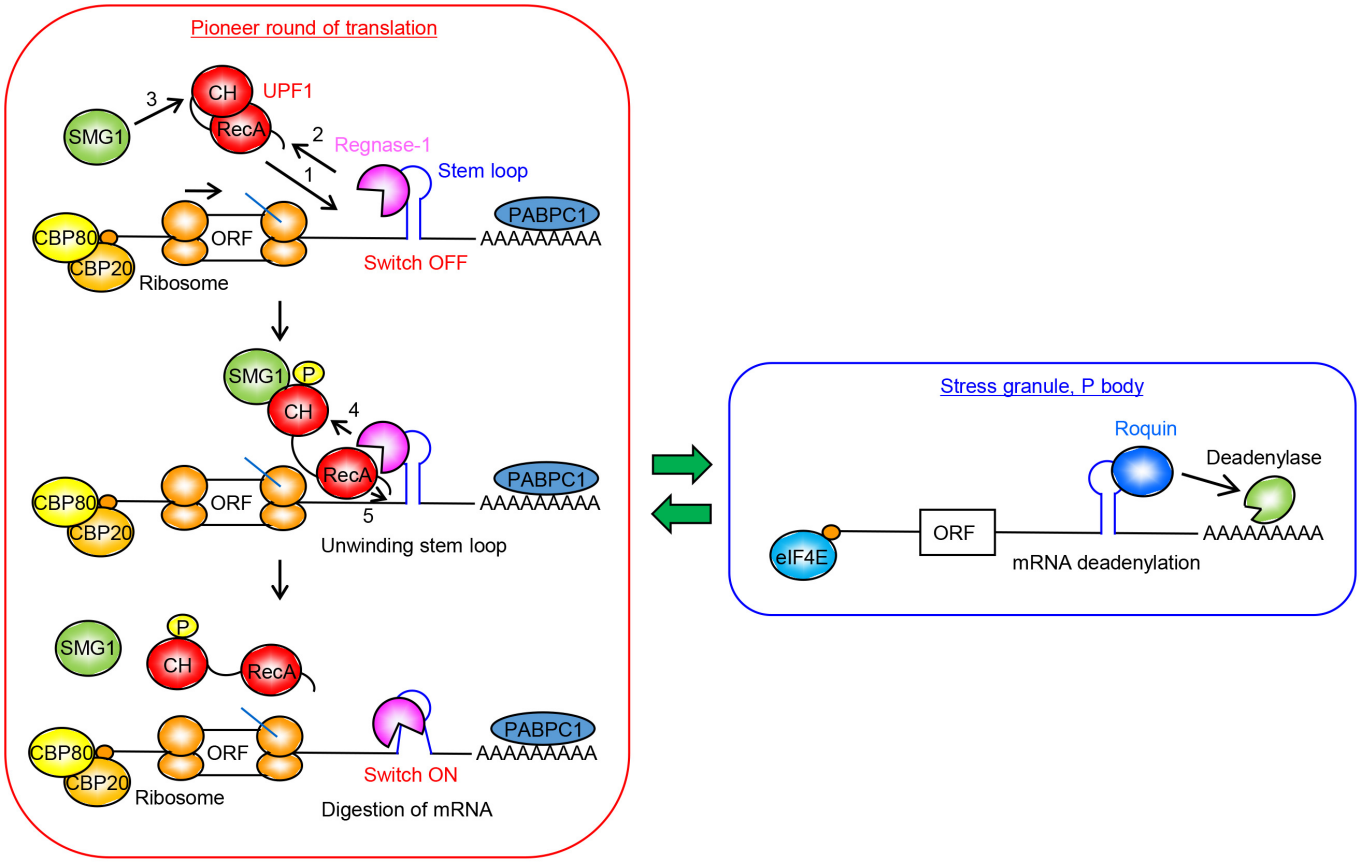
A proposed model of mRNA degradation by Regnase-1 and Roquin. Regnase-1 recognizes stem–loop on 3′ UTR of inflammatory mRNAs prior to binding with UPF1 and translation. In the Regnase-1 pathway, SMG1 phosphorylates UPF1 at T28, which is supported by the association of Regnase-1-(90-130) linker region with UPF1 RecA helicase domain. The T28-phosphorylated N-terminal region of UPF1 is associated with Regnase-1 RNase domain (K257R258) to form stable interaction between Regnase-1 and UPF1. The association with Regnase-1 promotes UPF1 ATPase/helicase activity and the unwinding of stem–loop by UPF1 helicase activity induces RNA cleavage by Regnase-1. The SMG1-UPF1–Regnase-1 axis destabilizes inflammatory mRNAs bound by CBP80 during pioneer round of translation, whereas Roquin regulates mRNAs bound by eIF4E after steady-state round of translation. NCR, N-terminal conserved region; RecA, RecA-like domain.

In this study, multiple lines of evidence suggest that translation termination induces unwinding of Regnase-1 target stem–loop RNAs by UPF1, after which RMD proceeds (Figure 8). Previously described mechanisms of gene regulation by mRNA involve recruitment of RNases to target mRNAs, or dimerization of the RNases to activate RNase activity. To our knowledge, structural changes in the target RNA itself that act as a molecular switch to activate RNA degradation have not been reported. The interaction between Regnase-1 with UPF1 described here explains the role of the stem–loop structure in both recruiting Regnase-1 and in regulating mRNA degradation during a pioneer round of translation.

Recent studies imply that endoribonucleases often collaborate with RNA helicases. This is exemplified by the relationship between SMG6 and UPF1 in NMD. The mechanism triggering RMD is reminiscent of NMD, in which UPF1 ATPase/helicase activity is essential. However, there is a clear difference between NMD and RMD. It was reported that UPF1 is required for the recruitment of SMG6 to NMD-targeted mRNAs by protein-protein interactions. Although UPF1 ATPase activity is required for mRNP disassembly and target discrimination in the NMD system, the requirement of UPF1 ATPase/helicase activity for NMD mRNA cleavage is obscure (24,25). On the other hand, UPF1 helicase-based remodeling RNA structure is directly required for initiation of RMD. Therefore, RMD possesses a heretofore undescribed mechanism by which RNA structural changes induced by UPF1 serve as a switch to activate Regnase-1.

A related example is an endoribonuclease Zucchini and an RNA helicase Armitage in the generation of piRNAs (52,53). Both Zucchini and Armitage are essential for piRNA biogenesis, but the molecular mechanism of Armitage- and Zucchini-mediated endoribonucleolytic cleavage has yet to be clarified. We speculate that RNA structural changes that act as a molecular switch for RNA cleavage are utilized by various endoribonucleases, including Zuccini-Armitage axis.

It is also known that RNA secondary structures can serve as the barrier for RNA degradation by non-specific exoribonucleases, and that RNA helicases in close association with non-specific RNases (for example, PNPase and RNase E) enhance the degradation of structured RNA (54). On the other hand, previously described sequence-specific or sequence-limited RNases (for example, RNase III family, RNase H, RNase A or RNase T1) do not require RNA structural changes to induce specific RNA cleavage (55). Thus, RNases might have developed multiple mechanisms to regulate degradation of target RNAs.

Considering the potentially deleterious effects of cytokine protein expression, the UPF1-mediated RNA structural change very likely functions as an essential timer for mRNA degradation by Regnase-1. This timing is achieved by the synergistic interaction between Regnase-1 and UPF1 following the pioneer round of translation, which, in turn, enhances the helicase activity of UPF1. We found that both the N-terminal region and RecA helicase domain of UPF1 are responsible for interaction between Regnase-1 and UPF1, whereas the C-terminal region of UPF1 functions as inhibitory domain for association with Regnase-1, suggesting that there is possibility that the C-terminal region of UPF1 inhibits the binding of Regnase-1 to the UPF1 N-terminal or central region. Future studies will reveal how the C-terminal region of UPF1 regulates RMD. Additionally, we found that the association between the disordered linker region of Regnase-1 and UPF1 RecA helicase domain is responsible for inducing UPF1 helicase activity by inhibiting intramolecular interactions with the CH domain. In NMD, it was reported that UPF2 associates with the CH-domain of UPF1 following the termination of translation (39,56), and subsequent conformational changes in UPF1 enhance the ATPase activity (39). On the other hand, Regnase-1 interacts with the RecA domain, but not the CH domain, of UPF1 via the Regnase-1-(90–130) linker region and UPF2 is dispensable for RMD (21). Future structural studies will uncover the mechanism by which Regnase-1 changes the conformation of UPF1, thus potentiating helicase activity by inhibiting the intramolecular interaction between the CH and RecA domains.

Consistent with these observations, we found that the phosphorylation of UPF1 at T28 is critical for stable interaction between Regnase-1 and UPF1 in cells. On the other hand, efficient degradation of target RNA by Regnase-1 was observed in the absence UPF1 phosphorylation or SMG1 *in vitro* system. Because high concentration of Regnase-1, UPF1 and target mRNA can induce the associations at high frequencies *in vitro*, RMD appears to be induced in the absence of UPF1 phosphorylation or SMG1 *in vitro*. In NMD, SMG6 is recruited to target mRNAs by the recognition of phospho-T28 of UPF1 via the 14-3-3-like domain (7). However, Regnase-1 does not harbor a 14-3-3-like domain; rather, the positively charged region in the RNase domain of Regnase-1 is responsible for the binding with the T28-phosphorylated UPF1. The interaction between the phospho-moiety and Regnase-1 RNase domain appears to be stable. Given the fact that recognition of phospho-T28 in UPF1 is essential for RMD, it is possible that the formation of a stable complex is critical for the execution of degradation by combining the UPF1 helicase and Regnase-1 RNase activities.

We identified SMG1 as a kinase that carries out UPF1 phosphorylation at the N-terminal end and is essential for Regnase-1 activity. In addition to NMD, UPF1 has been shown to be essential for translation-dependent mRNA degradation pathways such as Staufen-mediated decay, replication-dependent histone mRNA decay and maintenance of telomere length via the regulation of long noncoding telomeric repeat–containing RNA (TERRA) (46). However, other than the reported hyper-phosphorylation of UPF1 by ATR and DNA-dependent protein kinase in the previously described replication-dependent histone mRNA (57), it is not clear if UPF1 activity is generally regulated by its phosphorylation. In this regard, RMD appears to be more similar to NMD than to other UPF1-dependent RNA-degradation pathways.

Interestingly, T28-phosphorylation of UPF1 was rapidly induced in innate immune cells in response to TLR stimulation. One possible explanation for this is that an increase in Regnase-1 target mRNAs by TLR stimulation also induces the phosphorylation of UPF1. Alternatively, SMG1 kinase activity itself might be directly regulated in the course of the TLR signaling. Indeed, it is well known that the PI-3 kinase, which is related to SMG1, is activated in response to TLR ligands, and further activates downstream Akt/mTOR, thereby inhibiting TLR-induced inflammatory responses (58). SMG1 is reported to form a complex with SMG8 and SMG9, which suppress SMG1 kinase activity to modulate NMD (59,60), suggesting that SMG1 kinase activity can be regulated by modulating the SMG1 complex. Future studies will reveal if there are signaling pathways that directly control SMG1 phosphorylation of UPF1 in order to control immune cell activation.

In mice, SMG1 deficiency leads to embryonic lethality at day 8.5, due to impaired NMD (61). Nevertheless, *Smg1* gene-trap heterozygous (*Smg1^+/gt^*) mice showed increased production of cytokines, including Il6, and developed chronic inflammation (62). Surprisingly, NMD was not impaired in the *Smg1^+/gt^* mice irrespective of the severe defect in SMG1 expression. The phenotype is consistent with our finding that SMG1 regulates cytokine expression at the post-transcriptional level through the Regnase-1 pathway. Thus, the SMG1-UPF1–Regnase-1 pathway appears to be critical for cytokine expression *in vivo*.

We propose that Regnase-1 is essential for efficiently degrading inflammatory mRNAs undergoing pioneer rounds of translation. Although extensive studies showed that various RNA binding proteins such as ARE-BPs and Roquin recognize *cis*-elements present in the 3′ UTRs in inflammatory mRNAs in order to carry out degradation, to date none have implicated the importance of mRNA translation status. We found that RMD significantly restricts the number of proteins produced by an mRNA, and that this can be explained by our mathematical model in terms of regulation at the pioneer round of translation. This mechanism enables rapid degradation of inflammatory genes when the transcriptional supply ceases through clearance of inflammatory stimuli such as pathogens. A recent intriguing study shows that Regnase-1 functions to silence translation (63), though the molecular mechanism of how Regnase-1 inhibits protein synthesis is unclear. Our model indicates that Regnase-1 controls mRNAs undergoing initial rounds of translation, resulting in the decrease of protein production and protein/mRNA ratio. Thus, our model might also explain the function of Regnase-1 on the inhibition of translation.

Inhibition of SMG1 by a low molecular weight compound induced DC maturation by suppressing RMD. The compound, 11J, has been characterized as a highly specific SMG1 inhibitor that does not affect other PI-3 kinases (27). However, it was not known that SMG1 inhibition potentiates inflammation by suppressing RMD. Not only cytokine mRNAs, but also co-stimulatory molecules such as CD40 and CD80 expression, are up-regulated by lack of Regnase-1 or inhibition of SMG1. These co-stimulatory molecules are also directly regulated by Regnase-1 via their 3′ UTRs. Therefore, inhibition of SMG1 is an ideal way to potentiate innate immune cell activation, and a SMG1 inhibitor could be beneficial therapeutically.

In conclusion, this study provides a much clearer understanding of the synergistic relationship between Regnase-1, UPF1 and the stem–loop structures of RMD-targeted mRNAs. RNA structural changes play a critical role in a switch that controls their degradation. An understanding of post-transcriptional regulation of inflammatory genes requires consideration of pioneer rounds of translation as a critical regulation phase that is separate from regulation of steady-state translation. Specifically, the SMG1–UPF1–Regnase-1 pathway targets inflammatory mRNAs efficiently utilizes this phase. Reciprocally, manipulation of Regnase-1 activity via a SMG1 inhibitor is shown to potentiate innate immune cell activation. Thus, therapeutic targeting of the post-transcriptional regulation of inflammatory mRNAs could be beneficial for the development novel vaccine adjuvants.

## DATA AVAILABILITY

The RIP-seq data were deposited at DNA Data Bank of Japan (DDBJ) under the accession number DRA007389.

## Supplementary Material

gkz628_Supplemental_Files

## References

[1] Parker R., Song H. The enzymes and control of eukaryotic mRNA turnover. Nat. Struct. Mol. Biol. 2004; 11:121–127. 14749774 10.1038/nsmb724

[2] Schoenberg D.R., Maquat L.E. Regulation of cytoplasmic mRNA decay. Nat. Rev. Genet. 2012; 13:246–259. 22392217 10.1038/nrg3160 PMC3351101

[3] Anderson P. Post-transcriptional regulons coordinate the initiation and resolution of inflammation. Nat. Rev. Immunol. 2010; 10:24–35. 20029446 10.1038/nri2685

[4] Kafasla P., Skliris A., Kontoyiannis D.L. Post-transcriptional coordination of immunological responses by RNA-binding proteins. Nat. Immunol. 2014; 15:492–502. 24840980 10.1038/ni.2884

[5] Huntzinger E., Kashima I., Fauser M., Sauliere J., Izaurralde E. SMG6 is the catalytic endonuclease that cleaves mRNAs containing nonsense codons in metazoan. RNA. 2008; 14:2609–2617. 18974281 10.1261/rna.1386208 PMC2590965

[6] Eberle A.B., Lykke-Andersen S., Muhlemann O., Jensen T.H. SMG6 promotes endonucleolytic cleavage of nonsense mRNA in human cells. Nat. Struct. Mol. Biol. 2009; 16:49–55. 19060897 10.1038/nsmb.1530

[7] Okada-Katsuhata Y., Yamashita A., Kutsuzawa K., Izumi N., Hirahara F., Ohno S. N- and C-terminal Upf1 phosphorylations create binding platforms for SMG-6 and SMG-5:SMG-7 during NMD. Nucleic Acids Res. 2012; 40:1251–1266. 21965535 10.1093/nar/gkr791 PMC3273798

[8] Kim Y.K., Maquat L.E. UPFront and center in RNA decay: UPF1 in nonsense-mediated mRNA decay and beyond. RNA. 2019; 25:407–422. 30655309 10.1261/rna.070136.118 PMC6426291

[9] Kurosaki T., Popp M.W., Maquat L.E. Quality and quantity control of gene expression by nonsense-mediated mRNA decay. Nat. Rev. Mol. Cell Biol. 2019; 20:406–420. 30992545 10.1038/s41580-019-0126-2 PMC6855384

[10] Zhang K., Kaufman R.J. From endoplasmic-reticulum stress to the inflammatory response. Nature. 2008; 454:455–462. 18650916 10.1038/nature07203 PMC2727659

[11] Lavoie H., Li J.J., Thevakumaran N., Therrien M., Sicheri F. Dimerization-induced allostery in protein kinase regulation. Trends Biochem. Sci. 2014; 39:475–486. 25220378 10.1016/j.tibs.2014.08.004

[12] Ali M.M., Bagratuni T., Davenport E.L., Nowak P.R., Silva-Santisteban M.C., Hardcastle A., McAndrews C., Rowlands M.G., Morgan G.J., Aherne W. et al. Structure of the Ire1 autophosphorylation complex and implications for the unfolded protein response. EMBO J. 2011; 30:894–905. 21317875 10.1038/emboj.2011.18 PMC3049214

[13] Han Y., Donovan J., Rath S., Whitney G., Chitrakar A., Korennykh A. Structure of human RNase L reveals the basis for regulated RNA decay in the IFN response. Science. 2014; 343:1244–1248. 24578532 10.1126/science.1249845 PMC4731867

[14] Huang H., Zeqiraj E., Dong B., Jha B.K., Duffy N.M., Orlicky S., Thevakumaran N., Talukdar M., Pillon M.C., Ceccarelli D.F. et al. Dimeric structure of pseudokinase RNase L bound to 2–5A reveals a basis for interferon-induced antiviral activity. Mol. Cell. 2014; 53:221–234. 24462203 10.1016/j.molcel.2013.12.025 PMC3974923

[15] Wan Y., Kertesz M., Spitale R.C., Segal E., Chang H.Y. Understanding the transcriptome through RNA structure. Nat. Rev. Genet. 2011; 12:641–655. 21850044 10.1038/nrg3049 PMC3858389

[16] Mizrahi O., Nachshon A., Shitrit A., Gelbart I.A., Dobesova M., Brenner S., Kahana C., Stern-Ginossar N. Virus-Induced changes in mRNA secondary structure uncover cis-Regulatory elements that directly control gene expression. Mol. Cell. 2018; 72:862–874. 30318442 10.1016/j.molcel.2018.09.003

[17] Matsushita K., Takeuchi O., Standley D.M., Kumagai Y., Kawagoe T., Miyake T., Satoh T., Kato H., Tsujimura T., Nakamura H. et al. Zc3h12a is an RNase essential for controlling immune responses by regulating mRNA decay. Nature. 2009; 458:1185–1190. 19322177 10.1038/nature07924

[18] Yoshinaga M., Nakatsuka Y., Vandenbon A., Ori D., Uehata T., Tsujimura T., Suzuki Y., Mino T., Takeuchi O. Regnase-1 maintains iron homeostasis via the degradation of transferrin receptor 1 and prolyl-hydroxylase-dDomain-containing protein 3 mRNAs. Cell Rep. 2017; 19:1614–1630. 28538180 10.1016/j.celrep.2017.05.009

[19] Iwasaki H., Takeuchi O., Teraguchi S., Matsushita K., Uehata T., Kuniyoshi K., Satoh T., Saitoh T., Matsushita M., Standley D.M. et al. The IkappaB kinase complex regulates the stability of cytokine-encoding mRNA induced by TLR-IL-1R by controlling degradation of regnase-1. Nat. Immunol. 2011; 12:1167–1175. 22037600 10.1038/ni.2137

[20] Leppek K., Schott J., Reitter S., Poetz F., Hammond M.C., Stoecklin G. Roquin promotes constitutive mRNA decay via a conserved class of stem–loop recognition motifs. Cell. 2013; 153:869–881. 23663784 10.1016/j.cell.2013.04.016

[21] Mino T., Murakawa Y., Fukao A., Vandenbon A., Wessels H.H., Ori D., Uehata T., Tartey S., Akira S., Suzuki Y. et al. Regnase-1 and roquin regulate a common element in inflammatory mRNAs by spatiotemporally distinct mechanisms. Cell. 2015; 161:1058–1073. 26000482 10.1016/j.cell.2015.04.029

[22] Yu D., Tan A.H., Hu X., Athanasopoulos V., Simpson N., Silva D.G., Hutloff A., Giles K.M., Leedman P.J., Lam K.P. et al. Roquin represses autoimmunity by limiting inducible T-cell co-stimulator messenger RNA. Nature. 2007; 450:299–303. 18172933 10.1038/nature06253

[23] Jeltsch K.M., Hu D., Brenner S., Zoller J., Heinz G.A., Nagel D., Vogel K.U., Rehage N., Warth S.C., Edelmann S.L. et al. Cleavage of roquin and regnase-1 by the paracaspase MALT1 releases their cooperatively repressed targets to promote T(H)17 differentiation. Nat. Immunol. 2014; 15:1079–1089. 25282160 10.1038/ni.3008

[24] Franks T.M., Singh G., Lykke-Andersen J. Upf1 ATPase-dependent mRNP disassembly is required for completion of nonsense- mediated mRNA decay. Cell. 2010; 143:938–950. 21145460 10.1016/j.cell.2010.11.043 PMC3357093

[25] Lee S.R., Pratt G.A., Martinez F.J., Yeo G.W., Lykke-Andersen J. Target discrimination in nonsense-mediated mRNA decay requires Upf1 ATPase activity. Mol. Cell. 2015; 59:413–425. 26253027 10.1016/j.molcel.2015.06.036 PMC4673969

[26] Deans R.M., Morgens D.W., Okesli A., Pillay S., Horlbeck M.A., Kampmann M., Gilbert L.A., Li A., Mateo R., Smith M. et al. Parallel shRNA and CRISPR-Cas9 screens enable antiviral drug target identification. Nat. Chem. Biol. 2016; 12:361–366. 27018887 10.1038/nchembio.2050 PMC4836973

[27] Gopalsamy A., Bennett E.M., Shi M., Zhang W.G., Bard J., Yu K. Identification of pyrimidine derivatives as hSMG-1 inhibitors. Bioorg. Med. Chem. Lett. 2012; 22:6636–6641. 23021994 10.1016/j.bmcl.2012.08.107

[28] Yamashita A., Ohnishi T., Kashima I., Taya Y., Ohno S. Human SMG-1, a novel phosphatidylinositol 3-kinase-related protein kinase, associates with components of the mRNA surveillance complex and is involved in the regulation of nonsense-mediated mRNA decay. Genes Dev. 2001; 15:2215–2228. 11544179 10.1101/gad.913001 PMC312771

[29] Ohnishi T., Yamashita A., Kashima I., Schell T., Anders K.R., Grimson A., Hachiya T., Hentze M.W., Anderson P., Ohno S. Phosphorylation of hUPF1 induces formation of mRNA surveillance complexes containing hSMG-5 and hSMG-7. Mol. Cell. 2003; 12:1187–1200. 14636577 10.1016/s1097-2765(03)00443-x

[30] Wang D.O., Ninomiya K., Mori C., Koyama A., Haan M., Kitabatake M., Hagiwara M., Chida K., Takahashi S.I., Ohno M. et al. Transport granules bound with nuclear cap binding protein and exon junction complex are associated with microtubules and spatially separated from eIF4E granules and P bodies in human neuronal processes. Front. Mol. Biosci. 2017; 4:93. 29312956 10.3389/fmolb.2017.00093 PMC5744441

[31] Robinson M.D., McCarthy D.J., Smyth G.K. edgeR: a Bioconductor package for differential expression analysis of digital gene expression data. Bioinformatics. 2010; 26:139–140. 19910308 10.1093/bioinformatics/btp616 PMC2796818

[32] Subramanian A., Tamayo P., Mootha V.K., Mukherjee S., Ebert B.L., Gillette M.A., Paulovich A., Pomeroy S.L., Golub T.R., Lander E.S. et al. Gene set enrichment analysis: a knowledge-based approach for interpreting genome-wide expression profiles. Proc. Natl. Acad. Sci. U.S.A. 2005; 102:15545–15550. 16199517 10.1073/pnas.0506580102 PMC1239896

[33] Yamamoto S., De D., Hidaka K., Kim K.K., Endo M., Sugiyama H. Single molecule visualization and characterization of Sox2-Pax6 complex formation on a regulatory DNA element using a DNA origami frame. Nano Lett. 2014; 14:2286–2292. 24660747 10.1021/nl4044949

[34] Raz M.H., Hidaka K., Sturla S.J., Sugiyama H., Endo M. Torsional constraints of DNA substrates impact Cas9 cleavage. J. Am. Chem. Soc. 2016; 138:13842–13845. 27709924 10.1021/jacs.6b08915

[35] Shinohara H., Behar M., Inoue K., Hiroshima M., Yasuda T., Nagashima T., Kimura S., Sanjo H., Maeda S., Yumoto N. et al. Positive feedback within a kinase signaling complex functions as a switch mechanism for NF-kappaB activation. Science. 2014; 344:760–764. 24833394 10.1126/science.1250020

[36] Trott O., Olson A.J. AutoDock Vina: improving the speed and accuracy of docking with a new scoring function, efficient optimization, and multithreading. J. Comput. Chem. 2010; 31:455–461. 19499576 10.1002/jcc.21334 PMC3041641

[37] Gfeller D., Michielin O., Zoete V. SwissSidechain: a molecular and structural database of non-natural sidechains. Nucleic Acids Res. 2013; 41:D327–D332. 23104376 10.1093/nar/gks991 PMC3531096

[38] Wilamowski M., Gorecki A., Dziedzicka-Wasylewska M., Jura J. Substrate specificity of human MCPIP1 endoribonuclease. Sci. Rep. 2018; 8:7381. 29743536 10.1038/s41598-018-25765-2 PMC5943514

[39] Chakrabarti S., Jayachandran U., Bonneau F., Fiorini F., Basquin C., Domcke S., Le Hir H., Conti E. Molecular mechanisms for the RNA-dependent ATPase activity of Upf1 and its regulation by Upf2. Mol. Cell. 2011; 41:693–703. 21419344 10.1016/j.molcel.2011.02.010

[40] Matsuoka S., Ballif B.A., Smogorzewska A., McDonald E.R. 3rd, Hurov K.E., Luo J., Bakalarski C.E., Zhao Z., Solimini N., Lerenthal Y. et al. ATM and ATR substrate analysis reveals extensive protein networks responsive to DNA damage. Science. 2007; 316:1160–1166. 17525332 10.1126/science.1140321

[41] Kurosaki T., Li W., Hoque M., Popp M.W., Ermolenko D.N., Tian B., Maquat L.E. A post-translational regulatory switch on UPF1 controls targeted mRNA degradation. Genes Dev. 2014; 28:1900–1916. 25184677 10.1101/gad.245506.114 PMC4197951

[42] Durand S., Franks T.M., Lykke-Andersen J. Hyperphosphorylation amplifies UPF1 activity to resolve stalls in nonsense-mediated mRNA decay. Nat. Commun. 2016; 7:12434. 27511142 10.1038/ncomms12434 PMC4987530

[43] Yokogawa M., Tsushima T., Noda N.N., Kumeta H., Enokizono Y., Yamashita K., Standley D.M., Takeuchi O., Akira S., Inagaki F. Structural basis for the regulation of enzymatic activity of Regnase-1 by domain-domain interactions. Sci. Rep. 2016; 6:22324. 26927947 10.1038/srep22324 PMC4772114

[44] Izumi N., Yamashita A., Ohno S. Integrated regulation of PIKK-mediated stress responses by AAA+ proteins RUVBL1 and RUVBL2. Nucleus. 2012; 3:29–43. 22540023 10.4161/nucl.18926 PMC3337166

[45] Morita T., Yamashita A., Kashima I., Ogata K., Ishiura S., Ohno S. Distant N- and C-terminal domains are required for intrinsic kinase activity of SMG-1, a critical component of nonsense-mediated mRNA decay. J. Biol. Chem. 2007; 282:7799–7808. 17229728 10.1074/jbc.M610159200

[46] Isken O., Maquat L.E. The multiple lives of NMD factors: balancing roles in gene and genome regulation. Nat. Rev. Genet. 2008; 9:699–712. 18679436 10.1038/nrg2402 PMC3711694

[47] Maquat L.E., Tarn W.Y., Isken O. The pioneer round of translation: features and functions. Cell. 2010; 142:368–374. 20691898 10.1016/j.cell.2010.07.022 PMC2950652

[48] Rufener S.C., Muhlemann O. eIF4E-bound mRNPs are substrates for nonsense-mediated mRNA decay in mammalian cells. Nat. Struct. Mol. Biol. 2013; 20:710–717. 23665581 10.1038/nsmb.2576

[49] Durand S., Lykke-Andersen J. Nonsense-mediated mRNA decay occurs during eIF4F-dependent translation in human cells. Nat. Struct. Mol. Biol. 2013; 20:702–709. 23665580 10.1038/nsmb.2575

[50] Vinuesa C.G., Cook M.C., Angelucci C., Athanasopoulos V., Rui L., Hill K.M., Yu D., Domaschenz H., Whittle B., Lambe T. et al. A RING-type ubiquitin ligase family member required to repress follicular helper T cells and autoimmunity. Nature. 2005; 435:452–458. 15917799 10.1038/nature03555

[51] Linterman M.A., Rigby R.J., Wong R.K., Yu D., Brink R., Cannons J.L., Schwartzberg P.L., Cook M.C., Walters G.D., Vinuesa C.G. Follicular helper T cells are required for systemic autoimmunity. J. Exp. Med. 2009; 206:561–576. 19221396 10.1084/jem.20081886 PMC2699132

[52] Nishimasu H., Ishizu H., Saito K., Fukuhara S., Kamatani M.K., Bonnefond L., Matsumoto N., Nishizawa T., Nakanaga K., Aoki J. et al. Structure and function of Zucchini endoribonuclease in piRNA biogenesis. Nature. 2012; 491:284–287. 23064230 10.1038/nature11509

[53] Rogers A.K., Situ K., Perkins E.M., Toth K.F. Zucchini-dependent piRNA processing is triggered by recruitment to the cytoplasmic processing machinery. Genes Dev. 2017; 31:1858–1869. 29021243 10.1101/gad.303214.117 PMC5695087

[54] Deutscher M.P. How bacterial cells keep ribonucleases under control. FEMS Microbiol. Rev. 2015; 39:350–361. 25878039 10.1093/femsre/fuv012 PMC4542689

[55] MacRae I.J., Doudna J.A. Ribonuclease revisited: structural insights into ribonuclease III family enzymes. Curr. Opin. Struct. Biol. 2007; 17:138–145. 17194582 10.1016/j.sbi.2006.12.002

[56] Kashima I., Yamashita A., Izumi N., Kataoka N., Morishita R., Hoshino S., Ohno M., Dreyfuss G., Ohno S. Binding of a novel SMG-1-Upf1-eRF1-eRF3 complex (SURF) to the exon junction complex triggers Upf1 phosphorylation and nonsense-mediated mRNA decay. Genes Dev. 2006; 20:355–367. 16452507 10.1101/gad.1389006 PMC1361706

[57] Choe J., Ahn S.H., Kim Y.K. The mRNP remodeling mediated by UPF1 promotes rapid degradation of replication-dependent histone mRNA. Nucleic Acids Res. 2014; 42:9334–9349. 25016523 10.1093/nar/gku610 PMC4132728

[58] Hamerman J.A., Pottle J., Ni M., He Y., Zhang Z.Y., Buckner J.H. Negative regulation of TLR signaling in myeloid cells–implications for autoimmune diseases. Immunol. Rev. 2016; 269:212–227. 26683155 10.1111/imr.12381 PMC4703580

[59] Yamashita A., Izumi N., Kashima I., Ohnishi T., Saari B., Katsuhata Y., Muramatsu R., Morita T., Iwamatsu A., Hachiya T. et al. SMG-8 and SMG-9, two novel subunits of the SMG-1 complex, regulate remodeling of the mRNA surveillance complex during nonsense-mediated mRNA decay. Genes Dev. 2009; 23:1091–1105. 19417104 10.1101/gad.1767209 PMC2682953

[60] Arias-Palomo E., Yamashita A., Fernandez I.S., Nunez-Ramirez R., Bamba Y., Izumi N., Ohno S., Llorca O. The nonsense-mediated mRNA decay SMG-1 kinase is regulated by large-scale conformational changes controlled by SMG-8. Genes Dev. 2011; 25:153–164. 21245168 10.1101/gad.606911 PMC3022261

[61] McIlwain D.R., Pan Q., Reilly P.T., Elia A.J., McCracken S., Wakeham A.C., Itie-Youten A., Blencowe B.J., Mak T.W. Smg1 is required for embryogenesis and regulates diverse genes via alternative splicing coupled to nonsense-mediated mRNA decay. Proc. Natl. Acad. Sci. U.S.A. 2010; 107:12186–12191. 20566848 10.1073/pnas.1007336107 PMC2901484

[62] Roberts T.L., Ho U., Luff J., Lee C.S., Apte S.H., MacDonald K.P., Raggat L.J., Pettit A.R., Morrow C.A., Waters M.J. et al. Smg1 haploinsufficiency predisposes to tumor formation and inflammation. Proc. Natl. Acad. Sci. U.S.A. 2013; 110:E285–E294. 23277562 10.1073/pnas.1215696110 PMC3557096

[63] Behrens G., Winzen R., Rehage N., Dorrie A., Barsch M., Hoffmann A., Hackermuller J., Tiedje C., Heissmeyer V., Holtmann H. A translational silencing function of MCPIP1/Regnase-1 specified by the target site context. Nucleic Acids Res. 2018; 46:4256–4270. 29471506 10.1093/nar/gky106 PMC5934641

