# Supplementary material for: Translation-dependent unwinding of stem–loops by UPF1 licenses Regnase-1 to degrade inflammatory mRNAs
Source: Nucleic Acids Res. 2019 Jul 22;47(16):8838–59. doi: 10.1093/nar/gkz628 (PMC7145602; doi:10.1093/nar/gkz628)
Supplement: gkz628_Supplemental_Files [file gkz628_supplemental_files.zip › Mino et al. Supplementary Figures and Tables-revised.pdf]

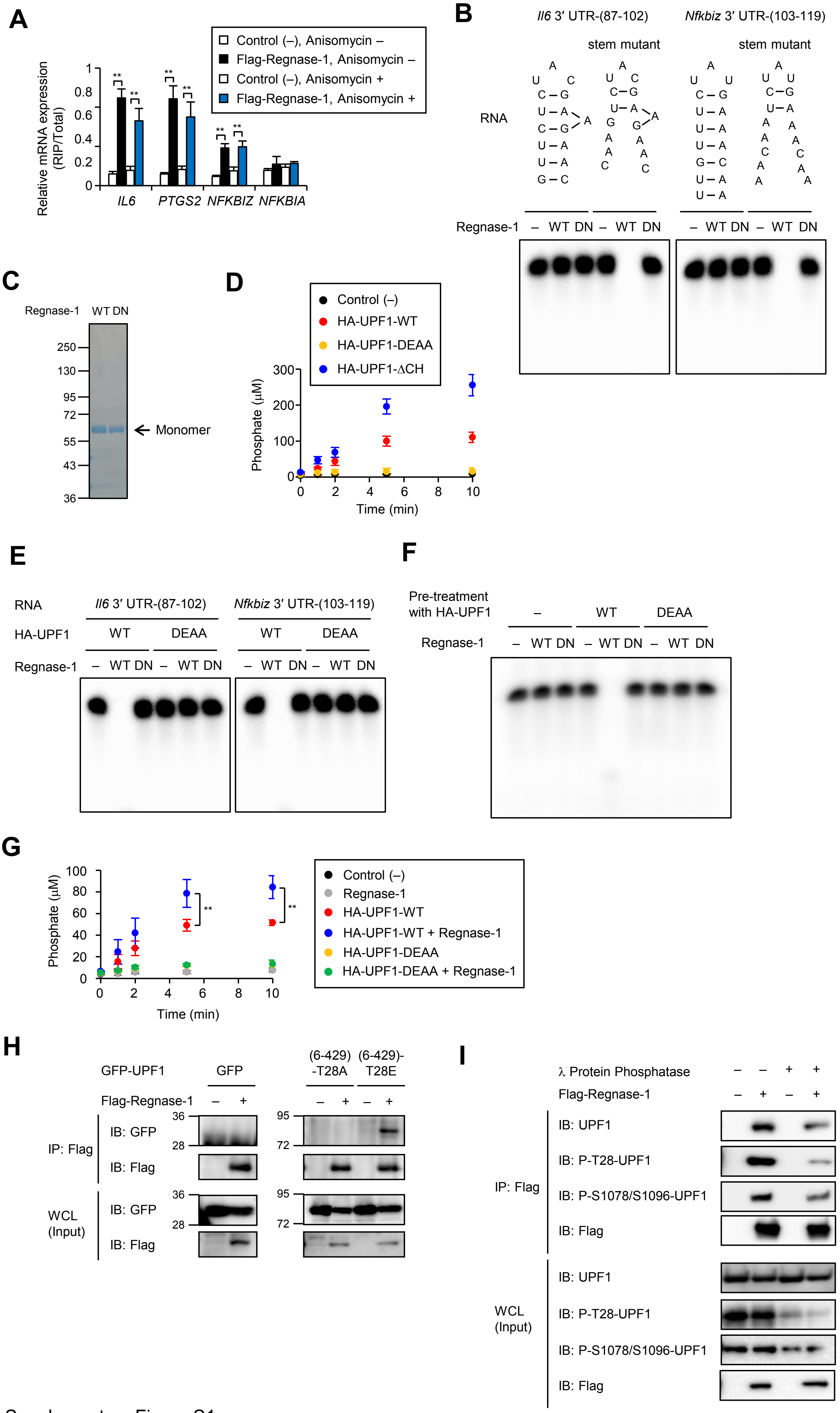

Supplementary Figure S1.

Supplementary Figure S1. The unwinding of stem-loop by RNA helicase UPF1 induces RNA cleavage by Regnase-1, Related to Figures 1 to 4

(A) mRNAs associated with Regnase-1 in HeLa cells transfected with indicated expression plasmids, stimulated with IL-1β (10 ng/mL) and treated with anisomycin (10 μg/mL) for 2 h were analyzed by RIP-qPCR.

(B) The stem-loop RNAs are not cleaved by Regnase-1 *in vitro* cleavage assay. Indicated 5′-^{32}P-labeled RNAs were incubated with Regnase-1 for 30 min, and analyzed by TBE-urea gel electrophoresis.

(C) Native PAGE analysis of Regnase-1.

(D) ATPase assay of HA-UPF1 in the presence of *Il6* 3′ UTR-(61-102) RNA.

(E) Regnase-1 cleaved the stem-loop RNAs in the presence of UPF1 *in vitro*. Indicated 5′-^{32}P-labeled RNAs were incubated with Regnase-1 and HA-UPF1 for 30 min, and analyzed by TBE-urea gel electrophoresis.

(F) Regnase-1 cleaved the *Il6* stem-loop RNA treated with UPF1 *in vitro*. The *Il6* stem-loop RNA treated with UPF1 were incubated with Regnase-1 for 30 min and analyzed by TBE-urea gel electrophoresis.

(G) ATPase assay of HA-UPF1 protein in the presence of Regnase-1.

(H) Western blot analysis of GFP-UPF1-(6-429)-T28A and -T28E mutant co-immunoprecipitated with Flag-Regnase-1 transiently-expressed in HeLa cells.

(I) Immunoblot analysis of UPF1 co-immunoprecipitated with Flag-Regnase-1 transiently-expressed in HeLa cells. Cell lysates were treated with λ protein phosphatase (400 U/mL) for 30 min at 30°C before co-immunoprecipitation.

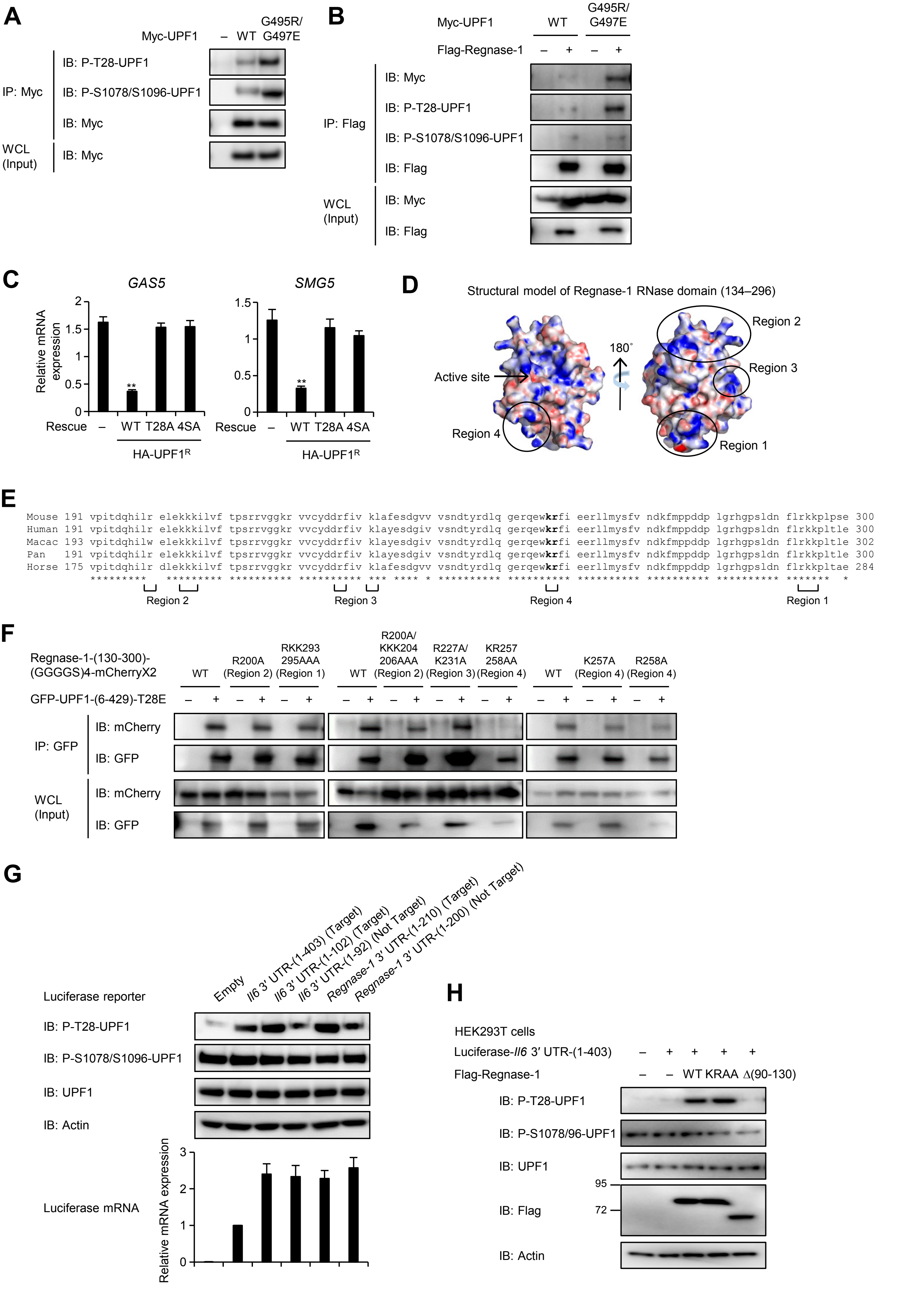

Supplementary Figure S2.

Supplementary Figure S2. The RNase domain of Regnase-1 interacts with T28-phosphorylated UPF1, Related to Figure 4

(A) Western blot analysis of P-T28-UPF1 and P-S1078/S1096-UPF1 in Myc-UPF1 and Myc-UPF1-G495R/G497E transiently-expressed in HeLa cells.

(B) Immunoblot analysis of Myc-UPF1, Myc-UPF1-G495R/G497E, P-T28-UPF1 and P-S1078/S1096-UPF1 co-immunoprecipitated with Flag-Regnase-1 transiently-expressed in HeLa cells.

(C) RNA expression levels in HeLa cells transfected with siRNA specific for UPF1, reconstituted with siRNA-resistant WT UPF1 or indicated its mutants. The cells were stimulated with IL-1β (10 ng/ml).

(D) Structural model of mouse Regnase-1 RNase domain (134–296).

(E) Schematic representation of amino acid sequences of Regnase-1 RNase domain (191-300) together with an alignment of 5 mammalian sequences.

(F) Western blot analysis of indicated Regnase-1-mCherry mutants co-immunoprecipitated with GFP-UPF1-(6-429)-T28E transiently-expressed in HeLa cells.

(G) Immunoblot analysis of P-T28-UPF1 and P-S1078/S1096-UPF1 in HeLa cells transfected with indicated luciferase reporter plasmids. Luciferase reporter mRNA expression levels were analyzed by RT-qPCR.

(H) Western blot analysis of phosphorylation levels of UPF1 in HEK293T cells transiently-coexpressing the indicated Flag-Regnase-1 and its mutants with luciferase reporter gene containing *Il6* 3′ UTR-(1-403).

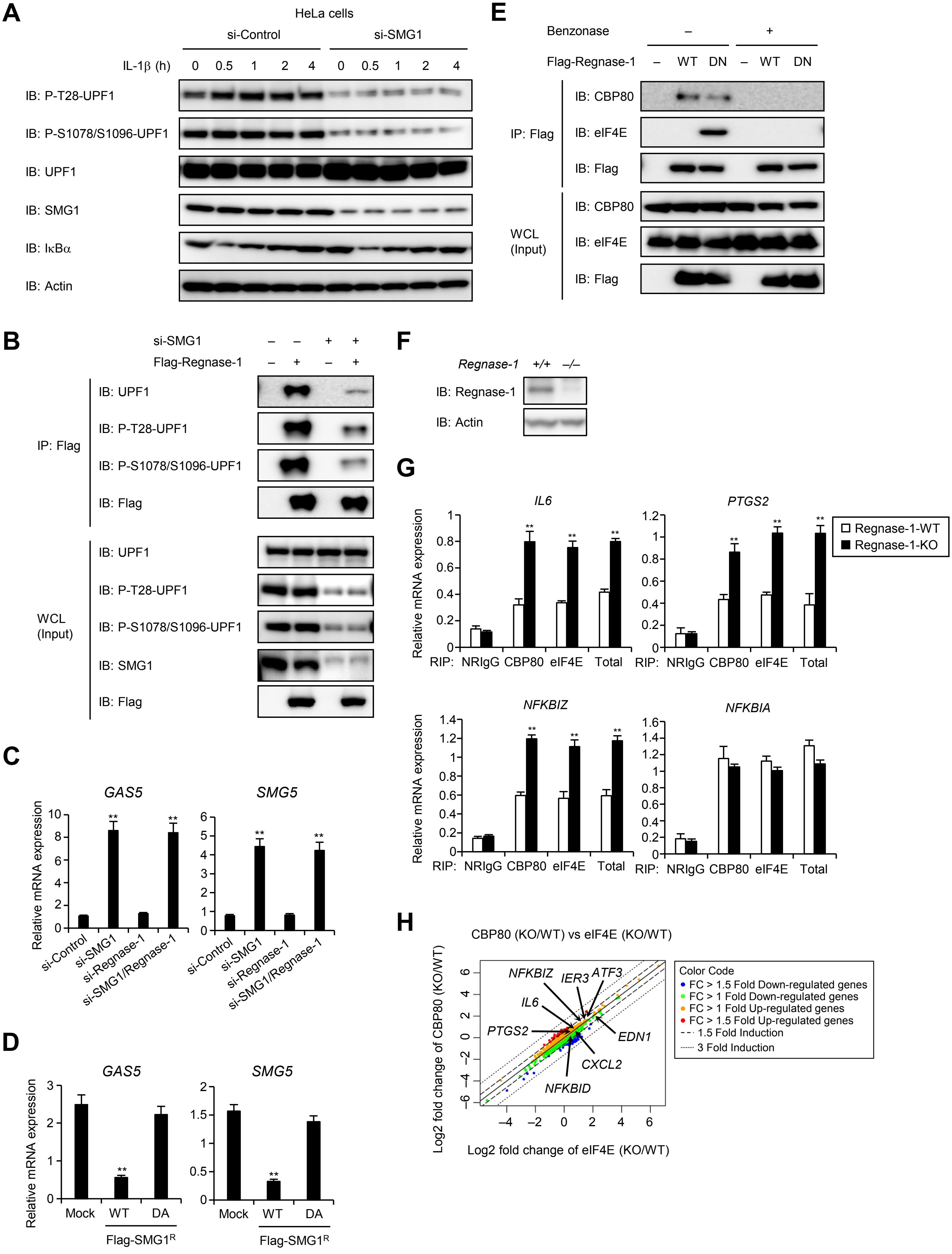
A
HeLa cells
si-Control
si-SMG1
IL-1β (h)
0 0.5 1 2 4 0 0.5 1 2 4
IB: P-T28-UPF1
IB: P-S1078/S1096-UPF1
IB: UPF1
IB: SMG1
IB: IκBα
IB: Actin
E
Benzonase – +
Flag-Regnase-1 – WT DN – WT DN
IP: Flag
IB: CBP80
IB: eIF4E
IB: Flag
WCL (Input)
IB: CBP80
IB: eIF4E
IB: Flag
B
si-SMG1 – – + +
Flag-Regnase-1 – + – +
IP: Flag
IB: UPF1
IB: P-T28-UPF1
IB: P-S1078/S1096-UPF1
IB: Flag
WCL (Input)
IB: UPF1
IB: P-T28-UPF1
IB: P-S1078/S1096-UPF1
IB: SMG1
IB: Flag
F
Regnase-1 +/+ –/–
IB: Regnase-1
IB: Actin
G
IL6
PTGS2
NFKBIZ
NFKBIA
Regnase-1-WT
Regnase-1-KO
Relative mRNA expression
RIP: NRIgG CBP80 eIF4E Total
C
GAS5
SMG5
Relative mRNA expression
si-Control
si-SMG1
si-Regnase-1
si-SMG1/Regnase-1
D
GAS5
SMG5
Relative mRNA expression
Mock WT DA
Flag-SMG1R
H
CBP80 (KO/WT) vs eIF4E (KO/WT)
Log2 fold change of CBP80 (KO/WT)
Log2 fold change of eIF4E (KO/WT)
NFKBIZ
IER3
ATF3
IL6
PTGS2
EDN1
CXCL2
NFKBID
Color Code
FC > 1.5 Fold Down-regulated genes
FC > 1 Fold Down-regulated genes
FC > 1 Fold Up-regulated genes
FC > 1.5 Fold Up-regulated genes
1.5 Fold Induction
3 Fold Induction

Figure S3.

Supplementary Figure S3. SMG1 regulates the interaction between Regnase-1 and UPF1, Related to Figure 5

(A)) Immunoblot analysis of P-T28-UPF1 and P-S1078/S1096-UPF1 in HeLa cells. HeLa cells were transfected with siRNA specific for SMG1 and stimulated with IL-1β (10 ng/mL).
(B) Western blot analysis of UPF1 co-immunoprecipitated with Flag-Regnase-1 transiently-expressed in HeLa cells under knockdown of SMG1.
(C) RNA expression levels in HeLa cells transfected with indicated siRNA and stimulated with IL-1β (10 ng/mL).
(D) RNA expression levels in HeLa cells transfected with siRNA specific for SMG1, reconstituted with siRNA-resistant WT or DA mutant of SMG1 and then stimulated with IL-1β (10 ng/ml).
(E) Western blot analysis of CBP80 and eIF4E co-immunoprecipitated with Flag-Regnase-1 in HeLa cells. Cell lysates were treated with Benzonase (50 U/mL) for 30 min at 25˚C before co-immunoprecipitation. Control immunoprecipitations were performed with normal rabbit IgG (NRIgG).
(F) Western blot analysis of Regnase-1 in HeLa cells. *Regnase-1*$^{-/-}$ (KO) HeLa cells were generated by CRISPR-Cas9 gene editing.
(G) RNA expression levels of CBP80- and eIF4E-associated mRNAs in Regnase-1-WT and –KO HeLa cells stimulated with IL-1β (10 ng/mL) for 2 h. Whole-cell lysates (WCLs) were prepared and RNA-protein complexes (RNPs) were immunoprecipitated from the WCLs using anti-CBP80 or anti-eIF4E antibody. Control immunoprecipitations were performed with normal rabbit IgG (NRIgG). Relative mRNA levels, normalized to endogenous β-actin mRNA, were determined by RT-qPCR from WCLs or CBP80-or eIF4E-associated fractions.
(H) Scatter plot of fold-changes between Regnase-1 WT and KO in CBP80-RIP against eIF4E-RIP.

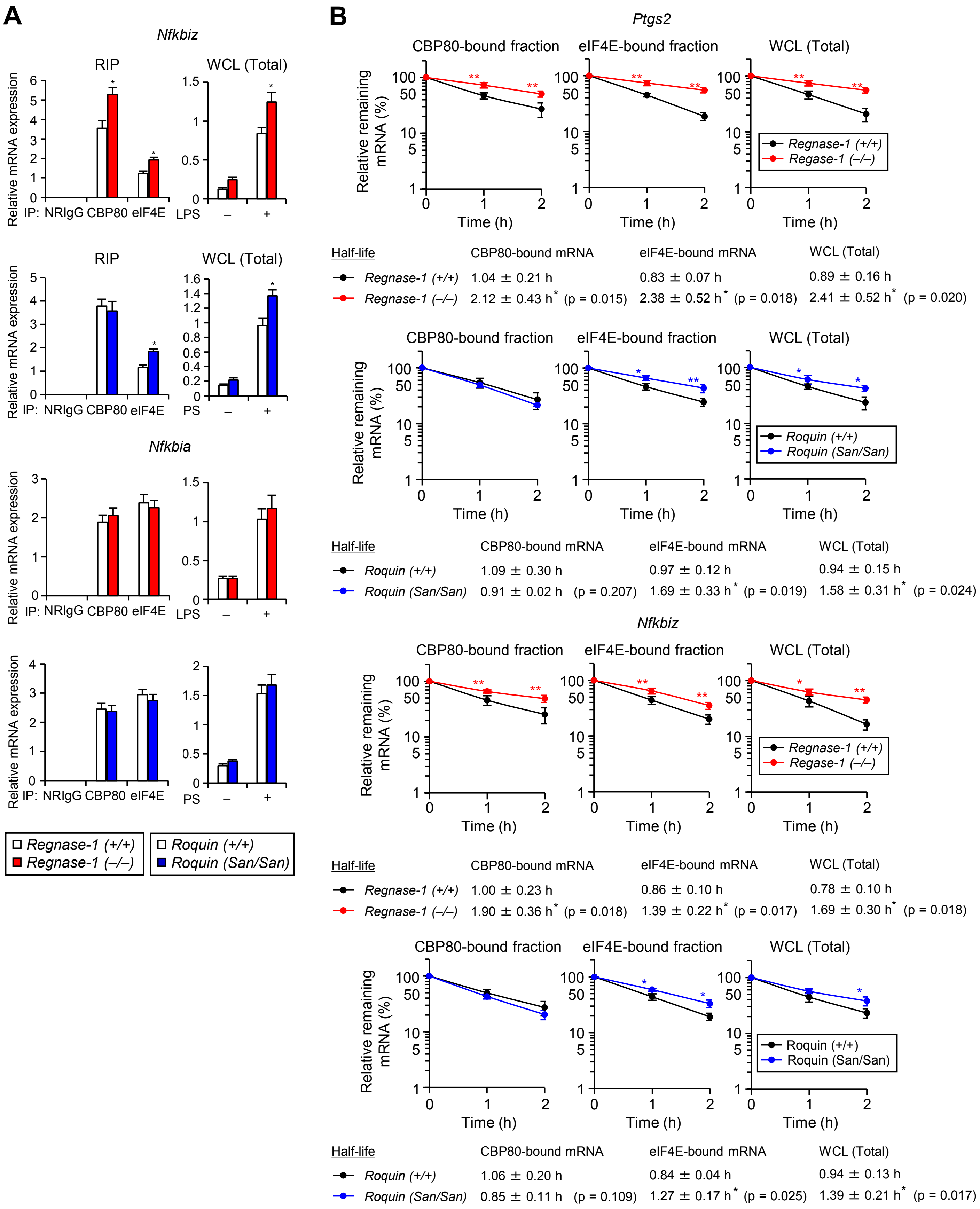

Supplementary Figure S4.

Supplementary Figure S4. Regnase-1 suppresses inflammatory mRNAs undergoing pioneer rounds of translation, Related to Figure 6

(A) RNA expression levels of CBP80- and eIF4E-associated mRNAs in MEFs stimulated with LPS (100 ng/mL) for 2 h. Whole-cell lysates (WCLs) were prepared and RNA-protein complexes (RNPs) were immunoprecipitated from the WCLs using anti-CBP80 or anti-eIF4E antibody. Control immunoprecipitations were performed with normal rabbit IgG (NRIgG). Relative mRNA levels, normalized to endogenous β-actin mRNA, were determined by RT-qPCR from WCLs or CBP80-or eIF4E-associated fractions.

(B) RNA degradation of CBP80- and eIF4E-associated mRNAs in MEFs stimulated with LPS (100 ng/mL) for 2 h and then treated with actinomycin D (1 μg/mL).

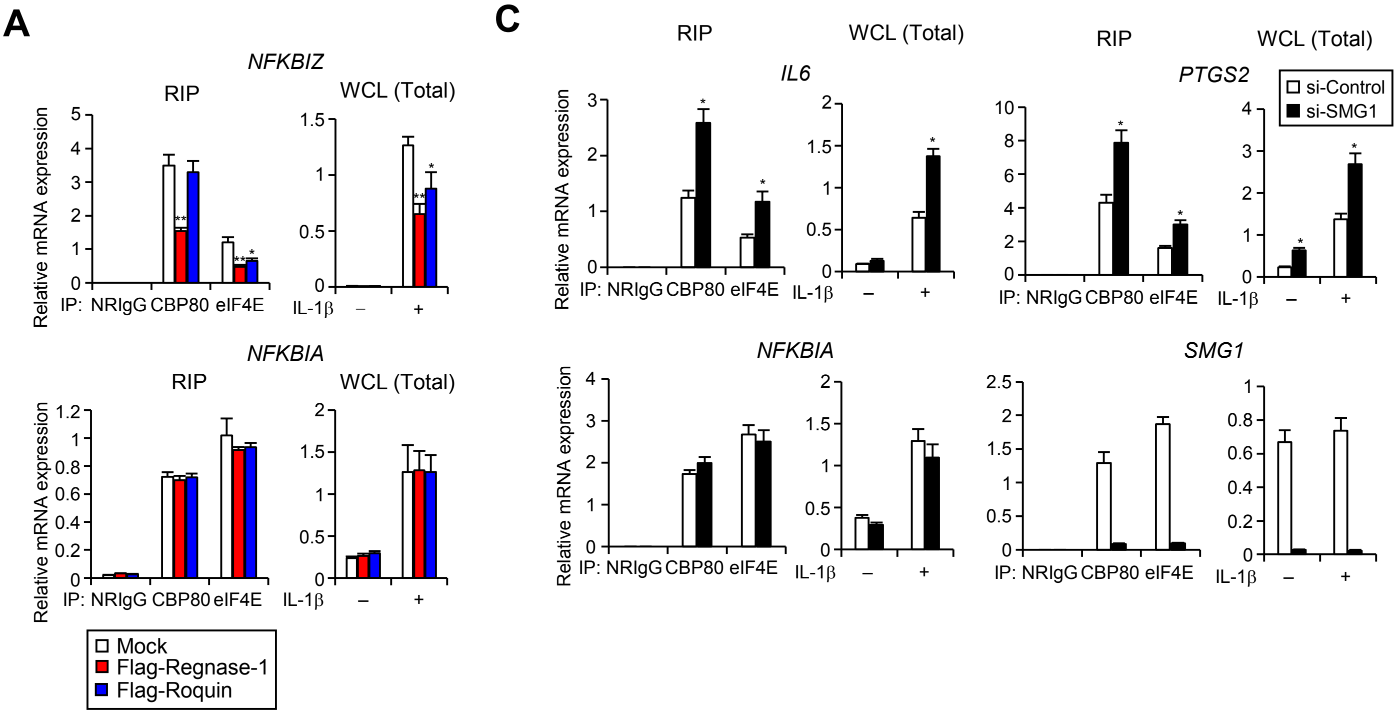

B

PTGS2

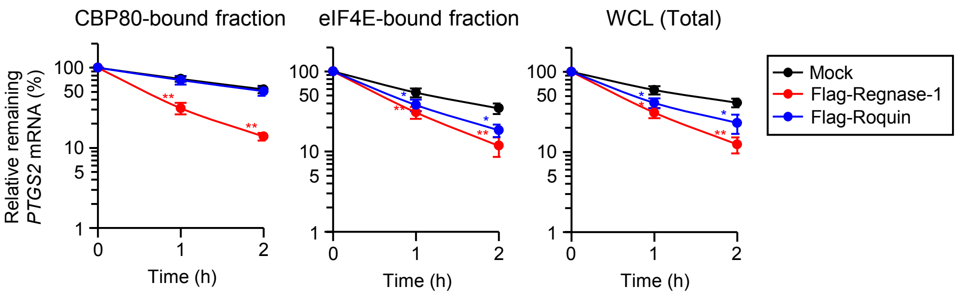

Half-life	CBP80-bound mRNA	eIF4E-bound mRNA	WCL (Total)
Mock	2.25 ± 0.41 h	1.28 ± 0.20 h	1.53 ± 0.29 h
Flag-Regnase-1	0.68 ± 0.05 h* (p = 0.023)	0.64 ± 0.08 h* (p = 0.014)	0.65 ± 0.07 h* (p = 0.037)
Flag-Roquin	2.07 ± 0.37 h	0.80 ± 0.09 h* (p = 0.032)	0.91 ± 0.16 h* (p = 0.049)

NFKBIZ

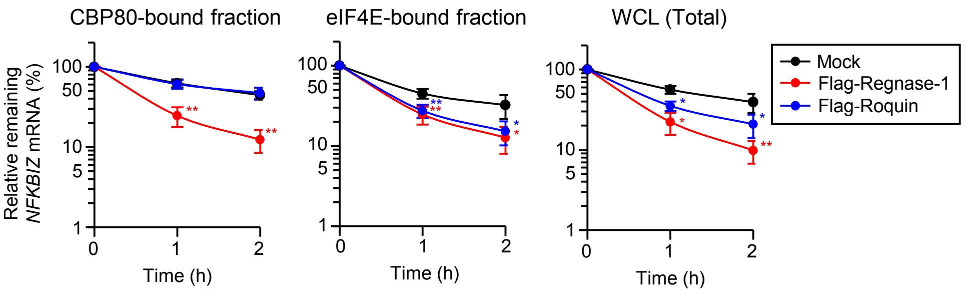

Half-life	CBP80-bound mRNA	eIF4E-bound mRNA	WCL (Total)
Mock	1.67 ± 0.27 h	1.16 ± 0.31 h	1.45 ± 0.36 h
Flag-Regnase-1	0.62 ± 0.10 h** (p = 0.0040)	0.63 ± 0.11 h* (p = 0.034)	0.56 ± 0.08 h* (p = 0.026)
Flag-Roquin	1.77 ± 0.42 h	0.69 ± 0.12 h* (p = 0.045)	0.83 ± 0.15 h* (p = 0.035)

Supplementary Figure S5.

Supplementary Figure S5. The SMG1-UPF1-Regnase-1 axis suppresses inflammatory mRNAs undergoing pioneer rounds of translation, Related to Figure 6

(A) RNA expression levels of CBP80- and eIF4E-associated mRNAs in HeLa cells transfected with the control (Mock), Flag-Regnase-1 or Flag-Roquin expression plasmid and stimulated with IL-1β (10 ng/mL) for 2 h.

(B) RNA degradation of CBP80- and eIF4E-associated mRNAs in HeLa cells transfected with the control (Mock), Flag-Regnase-1 or Flag-Roquin expression plasmid, stimulated with IL-1β (10 ng/mL) for 2 h and then treated with actinomycin D (1 μg/mL).

(C) RNA expression levels of CBP80- and eIF4E-associated mRNAs in HeLa cells transfected with siRNA specific for SMG1 and stimulated with IL-1β (10 ng/mL) for 2 h.

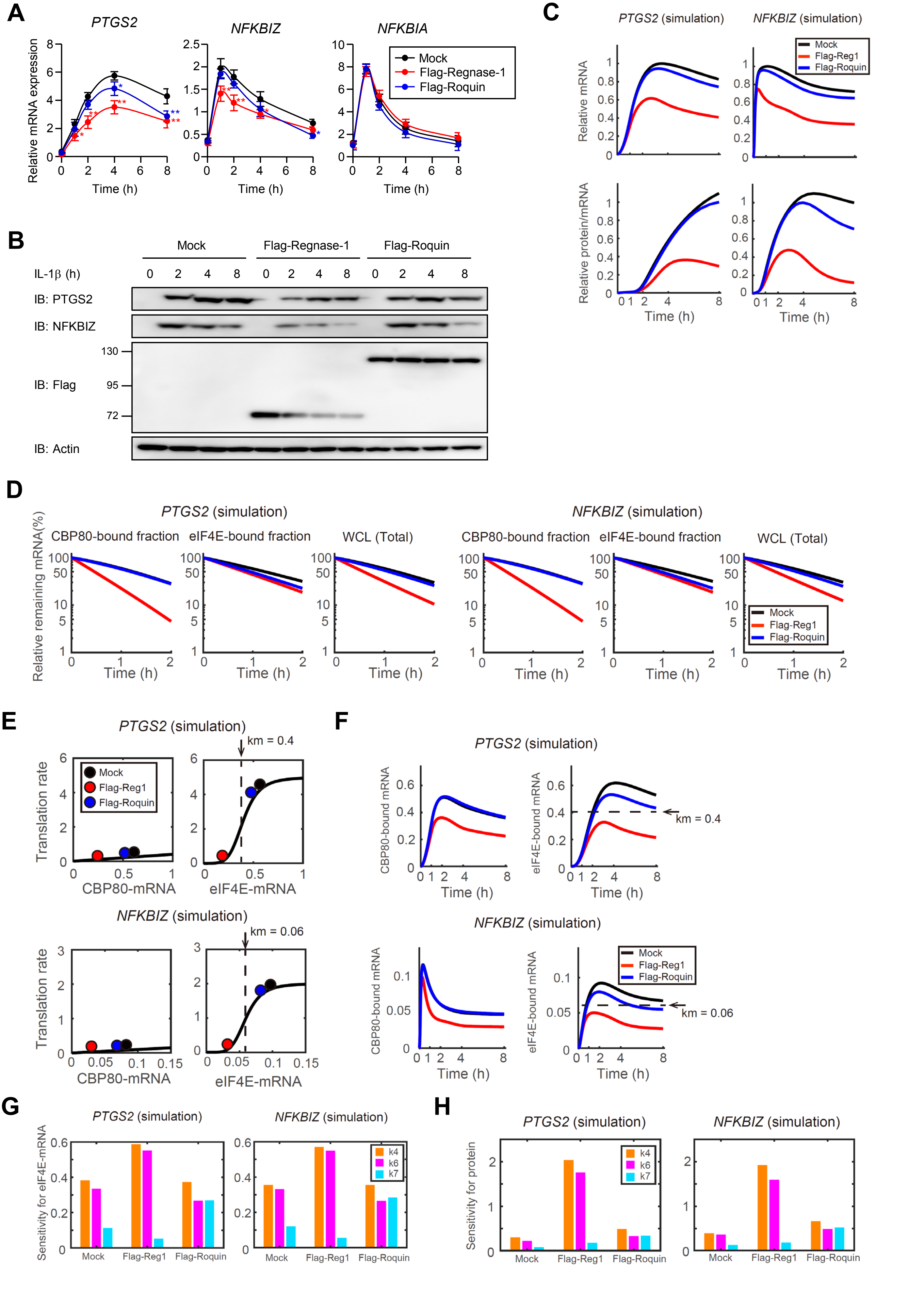

Supplementary Figure S6.

Supplementary Figure S6. Regnase-1 is more effective than Roquin in limitation of protein translation, Related to Figure 6

(A and B) HeLa cells were transfected with the control (Mock), Flag-Regnase-1 or Flag-Roquin expression plasmid and stimulated with IL-1β (10 ng/mL). RNA expression profiles were analyzed by RT-qPCR (A) and protein levels were measured by Western blotting (B).

(C) Simulation results of relative mRNA and protein/mRNA ratio of PTGS2 and NFKBIZ corresponding to Figures 6F and S6A. The simulation values were normalized with maximum values.

(D) Relative remaining mRNA after adding actinomycin D in simulation to Figures 1E and S1E. We set k1 = 0 and k2 = 0 in this simulation.

(E) Translation rates from CBP80-mRNA and eIF4E-mRNA for *PTGS2* and *NFKBIZ* in simulation. Circles indicate translation rates in Mock (black), Flag-Regnase-1 (red) and Flag-Roquin (blue) at 2 h after stimulation. Solid line indicates potential translation rates to the amounts of CBP80-bound mRNA and eIF4E-bound mRNA. km is maximal half effective concentration.

(F) CBP80-bound mRNA and eIF4E-bound mRNA of *PTGS2* and *NFKBIZ* in simulation.

(G) Sensitivity analysis of Regnase-1-induced degradation (k4, k6) and Roquin-induced degradation (k7) for eIF4E-mRNA.

(H) Sensitivity analysis for protein to Regnase-1-induced degradation (k4, k6) and Roquin-induced degradation (k7).

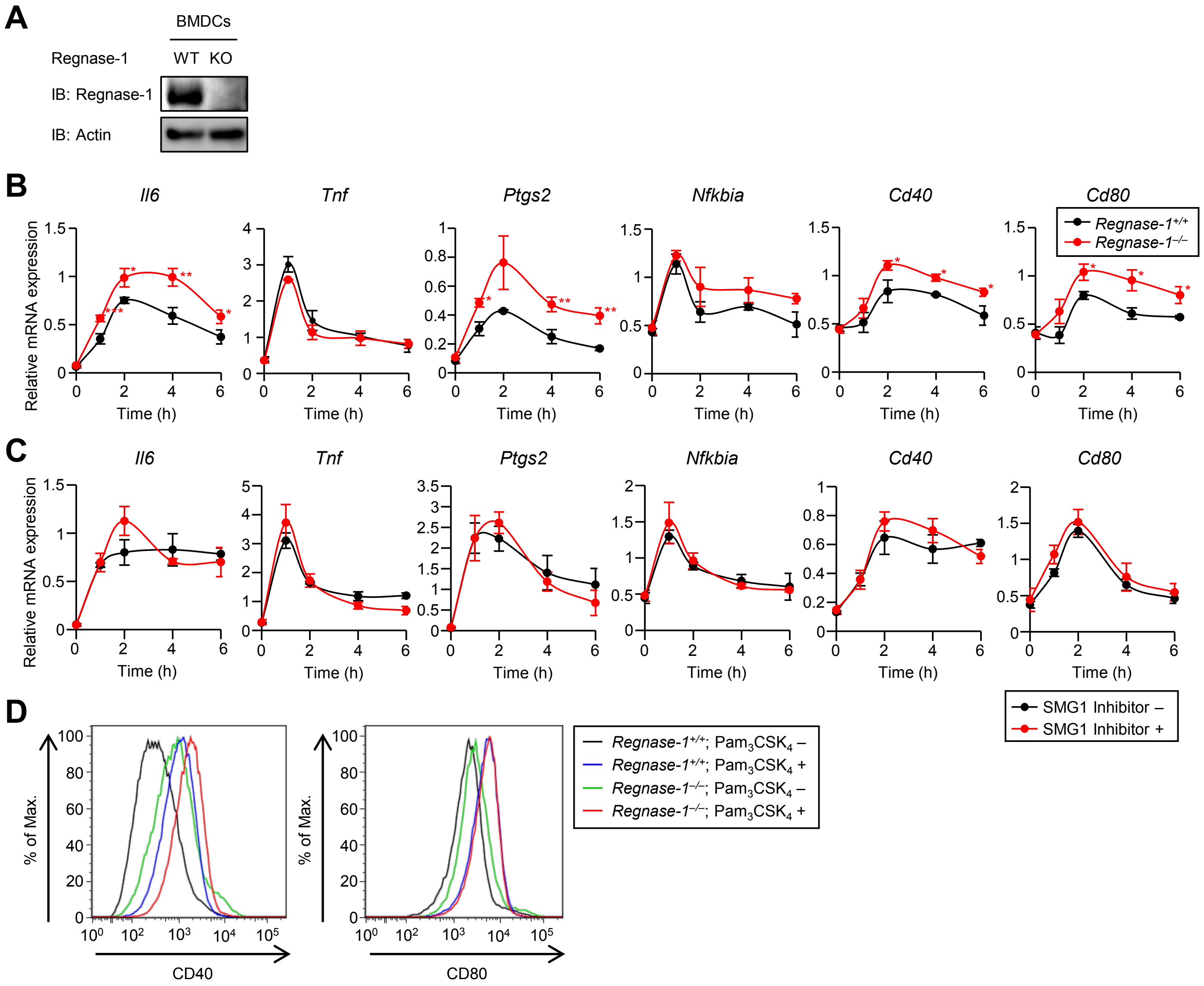

Supplementary Figure S7.

Supplementary Figure S7. Control of DC maturation and proinflammatory gene expression by manipulating SMG1 activity, Related to Figure 7

(A) Western blot analysis of Regnase-1 in BMDCs from *Regnase-1*$^{+/+}$ (WT) and *Regnase-1*$^{-/-}$ (KO) mice.

(B) RNA expression levels in BMDCs from *Regnase-1*$^{+/+}$ (WT) and *Regnase-1*$^{-/-}$ (KO) mice. The BMDCs were stimulated with Pam_3CSK_4 (1 ng/mL).

(C) RNA expression levels in BMDCs from *Regnase-1*$^{-/-}$ mice. The BMDCs were treated with SMG1 inhibitor (0.3 μM) and stimulated with Pam_3CSK_4 (1 ng/mL).

(D) BMDCs from *Regnase-1*$^{+/+}$ (WT) and *Regnase-1*$^{-/-}$ (KO) mice were cultured with Pam_3CSK_4 (1 ng/mL) for 48 hr. Expression of CD40 and CD80 on BMDCs were analyzed by Flow cytometry.

Supplementary Table S1. Parameters for PTGS2 production model.

ID	Value	Description of reaction rate
s_{input}	1	Parameter in signal
t_{pulse}	0	Parameter in signal
t_{raise}	1	Parameter in signal
t_{decay}	20	Parameter in signal
s_{base}	0	Parameter in signal
t_{delay}	0	Parameter in signal
s_{late}	0.1	Parameter in signal
k_1	1.0	Transcriptional rate
k_2	1.0	Transition of mRNA
k_3	0.1	Basal degradation of CmRNA
k_4	0.3	Reg1 induced degradation of CmRNA
k_5	0.3	Basal degradation of EmRNA
k_6	0.1	Reg1 induced degradation of EmRNA
k_7	0.1	Roq induced degradation of EmRNA
k_8	0.4	Translational rate from CmRNA
k_9	5.0	Translational rate from EmRNA
k_{10}	0.4	Degradation of protein
km	0.4	Half maximal effective conc.
h	5	Hill coefficient

Supplementary Table S2. Parameters for NFKBIZ production model.

ID	Value	Description of reaction rate
s_{input}	1	Parameter in signal
t_{pulse}	0	Parameter in signal
t_{raise}	0.1	Parameter in signal
t_{decay}	0.1	Parameter in signal
s_{base}	0	Parameter in signal
t_{delay}	0	Parameter in signal
s_{late}	0.1	Parameter in signal
k_1	1.0	Transcriptional rate
k_2	1.0	Transition of mRNA
k_3	0.1	Basal degradation of CmRNA
k_4	0.3	Reg1 induced degradation of CmRNA
k_5	0.3	Basal degradation of EmRNA
k_6	0.1	Reg1 induced degradation of EmRNA
k_7	0.1	Roq induced degradation of EmRNA
k_8	1.0	Translational rate from CmRNA
k_9	2.0	Translational rate from EmRNA
k_{10}	1.0	Degradation of protein
km	0.06	Half maximal effective conc.
h	5	Hill coefficient

Supplementary Table S3. Primers used for quantitative PCR analysis.

Gene	Sequence (5′ to 3′)
Mouse Il6 (Forward)	GTAGCTATGGTACTCCAGAAGAC
Mouse Il6 (Reverse)	ACGATGATGCACTTGCAGAA
Mouse Tnf (Forward)	CCCTCACACTCAGATCATCTTCT
Mouse Tnf (Reverse)	GCTACGACGTGGGCTACAG
Mouse Zc3h12a (Forward)	CGAGAGGCAGGAGTGGAAAC
Mouse Zc3h12a (Reverse)	CTTACGAAGGAAGTTGTCCAGGCTAG
Mouse Roquin (Rc3h1) (Forward)	GACCAGACCACTATCAATACGGAC
Mouse Roquin (Rc3h1) (Reverse)	CACTGCTGAGTGGCTTCAGGTAC
Mouse Roquin2 (Rc3h2) (Forward)	CACTAGGAGAAAGAACTGTGAC
Mouse Roquin2 (Rc3h2) (Reverse)	GCAGAACCATCTTCTAATGCCAGC
Mouse Nfkbiz (IkBz) (Forward)	CTCCGACTCCTCCGATTTCTC
Mouse Nfkbiz (IkBz) (Reverse)	GCTTGTTGCTTCGGATGTGTAG
Mouse Ptgs2 (Forward)	CTTGCTGTTCCAATCCATGTC
Mouse Ptgs2 (Reverse)	GTTCCAGACTCCCTTGAAGTG
Mouse Upf1 (Forward)	GCTGAACTTCGAGGAAGATG
Mouse Upf1 (Reverse)	CTTCCTTGCATTTTGCCCTC
Mouse Smg1 (Forward)	GTCTCGACTGTCGAATCTTC
Mouse Smg1 (Reverse)	CACATCATGAACAGCAGCCAAG
Mouse Nfkbia (IkBa) (Forward)	GAGGAGTACGAGCAAATGGTG
Mouse Nfkbia (IkBa) (Reverse)	CCTGACCAATGACTTCCATG
Mouse Gas5 (Forward)	CAGGTATTAATGGGTCACCTC
Mouse Gas5 (Reverse)	CTTCTATTTGAGCCTCCATCC
Mouse Smg5 (Forward)	CAGAGAACGTTAGCCTGAGG
Mouse Smg5 (Reverse)	CTGTAGGCACATTCCAAGGTG
Mouse β-Actin (Forward)	GGCTGTATTCCCCTCCATCG
Mouse β-Actin (Reverse)	CCAGTTGGTAACAATGCCATGT
Mouse Cd40 (Forward)	CTGTGAGGATAAGAACTTGGAGGTCC
Mouse Cd40 (Reverse)	GATGAGGATGCCCATCACGACAGG
Mouse Cd80 (Forward)	GACTCGCAACCACACCATTAAGTGTC
Mouse Cd80 (Reverse)	CCCCAAAGAGCACAAGTGTGTTC

Supplementary Table S3. Primers used for quantitative PCR analysis (continued).

Gene	Sequence (5′ to 3′)
Human IL6 (Forward)	CAGCCACTCACCTCTTCAGAAC
Human IL6 (Reverse)	GCATCCATCTTTTTCAGCCATCTTTGG
Human TNF (Forward)	CTGCCTGCTGCACTTTGGAGTG
Human TNF (Reverse)	CATTGGCCAGGAGGGCATTGG
Human PTGS2 (Forward)	CATGTCAAAACCGAGGTGTATG
Human PTGS2 (Reverse)	GAAGTGGGTAAGTATGTAGTGCAC
Human ZC3H12A (Forward)	GAAGAGGAAAAGGAGGGCAG
Human ZC3H12A (Reverse)	CTCCAGGATGGCACAAACAC
Human Roquin (RC3H1) (Forward)	GGTTGTGGCCATACTGTCTG
Human Roquin (RC3H1) (Reverse)	GCTTTGTGTCTTCAACCCCAC
Human Roquin2 (RC3H2) (Forward)	GACCAGACTGCCATCAACAC
Human Roquin2 (RC3H2) (Reverse)	GTAGAGTGCCAAATCCTCAACG
Human NFKBIZ (IkBz) (Forward)	GAAAGGGCCCGATTCGTTGTCTG
Human NFKBIZ (IkBz) (Reverse)	GAAGCAGATCAGCACTGCTCTC
Human UPF1 (Forward)	GTTGAACTTCGAGGAAGATG
Human UPF1 (Reverse)	CATTTTGCCCTCACAAGGTG
Human SMG1 (Forward)	CAGAGATAGAGGTGGTTCTTC
Human SMG1 (Reverse)	CATTGTTAACCCTCAGCTCTTG
Human NFKBIA (IkBa) (Forward)	GAGGAGTACGAGCAGATGGTC
Human NFKBIA (IkBa) (Reverse)	CAGGTTGTTCTGGAAGTTGAG
Human GAS5 (Forward)	GTGTGGCTCTGGATAGCACC
Human GAS5 (Reverse)	GAACCATTAAGCTGGTCCAGG
Human SMG5 (Forward)	GTGCATCGACTTGACCTCATC
Human SMG5 (Reverse)	GTATACCTTTCTCCACAGCAG
Human β-ACTIN (Forward)	CACCATTGGCAATGAGCGGTTCC
Human β-ACTIN (Reverse)	CTTCTGCATCCTGTCGGCAATGC

Supplementary Table S3. Primers used for the preparation of Northern blot probes (continued).

Gene	Sequence (5′ to 3′)
Mouse Il6 CDS (Forward)	GTTCCTCTCTGCAAGAGACTTCCATCC
Mouse Il6 CDS (Reverse)	GTATCTCTCTGAAGGACTCTGGCTTTG
Mouse β-Actin CDS (Forward)	CTATGTGGGTGACGAGGCCCAGAG
Mouse β-Actin CDS (Reverse)	GGGTACATGGTGGTACCACCAGAC
